# Seasonal asynchrony and harvest diversification contribute to demersal finfish fisheries stability in Chesapeake Bay

**DOI:** 10.1002/eap.70097

**Published:** 2025-09-11

**Authors:** Sean B. Hardison, Jonathan S. Lefcheck, Shelby B. White, Maowei Liang, Y. Stacy Zhang, Christopher J. Patrick, Andrew M. Scheld, Robert J. Latour, F. Joel Fodrie, Sean C. Anderson, Max C. N. Castorani

**Affiliations:** ^1^ Department of Environmental Sciences University of Virginia Charlottesville Virginia USA; ^2^ College of Fisheries and Ocean Sciences University of Alaska Fairbanks Juneau Alaska USA; ^3^ University of Maryland Center for Environmental Science Cambridge Maryland USA; ^4^ Virginia Institute of Marine Science William & Mary Gloucester Point Virginia USA; ^5^ Cedar Creek Ecosystem Science Reserve University of Minnesota East Bethel Minnesota USA; ^6^ Department of Marine, Earth and Atmospheric Sciences North Carolina State University Raleigh North Carolina USA; ^7^ Institute of Marine Sciences University of North Carolina at Chapel Hill Morehead City North Carolina USA; ^8^ Pacific Biological Station Fisheries and Oceans Canada Nanaimo British Columbia Canada

**Keywords:** asynchrony, commercial fisheries, long‐term ecological research, portfolio effects, social‐ecological system, spatiotemporal modeling, stability

## Abstract

Biodiversity can confer temporal stability to ecosystem processes through asynchrony in species' abundances and may promote asynchrony and stability of commercial fishing harvests derived from exploited species. However, the linkages between asynchrony in the population dynamics of commercially harvested species and asynchrony of associated harvests have been difficult to resolve due to ecological, social, and economic dynamics that mediate resource extraction. Here, we explored coupled human‐ecological relationships and emergent asynchrony using commercial fishing harvest data and fisheries‐independent trawl surveys in two regions (Maryland and Virginia) of Chesapeake Bay, USA, from 2002 to 2018. For each region, we sought to identify how seasonal (within‐year) asynchrony among harvested fish species contributed to (1) seasonal asynchrony in the harvests of these species and (2) within‐year stability and economic value of harvests. We found that, in Maryland, seasonal closure of striped bass (*Morone saxatilis*) fishing resulted in asynchrony by forcing switching to alternative stocks. In Virginia, seasonal migration of harvested species to and from the Chesapeake Bay promoted harvest compensation and therefore harvest asynchrony. However, this effect was negated by the concurrent effects of an increase in the evenness of species dynamics on harvest compensation, reflecting changes in fishing patterns, primarily following declines in the biomass of Atlantic croaker (*Micropogonias undulatus*). Our findings show that both social (direct management actions and behavioral responses) and emergent properties of ecological systems can influence asynchrony in dynamics of exploited populations and commercial harvests, with implications for their continued management and sustainability.

## INTRODUCTION

Effective management of social‐ecological systems is challenged by their immense complexity (Levin et al., 2013; Link, 2018). However, conserving and bolstering that complexity has long been promoted as a strategy to reduce variability in aggregate system properties, such as community biomass production (Tilman, 1996; Tilman et al., 1998) or total fishery harvest yield and revenue (Cline et al., 2017; Hilborn et al., 2001; Schindler et al., 2010). Consider, for instance, commercial fishing harvest portfolios, defined here as dynamic collections of harvests drawn from multiple species and/or populations that contribute to diverse ecological communities within a region (i.e., “stocks”). When commercial fishing portfolios are diversified across species, asynchronous harvest dynamics among species reduce portfolio variability (and enhance stability) over time relative to the average variability of the individual single‐species harvests, known as a “portfolio effect” (Anderson et al., 2017; Link, 2018). Despite the dependence of commercial fisheries on the population dynamics of targeted species, few studies have characterized the relationships between the asynchrony of populations of different species (species asynchrony) and the asynchrony of harvests drawn from those species (harvest asynchrony). Understanding the strength and directionality of these relationships can illuminate the stabilizing role of biodiversity in coupled social‐ecological systems, including commercial fisheries, which represent a large economic sector in many countries and provide food security to billions of people globally (FAO, 2024).

Although there are many ways to quantify asynchrony (Doak et al., 1998; Moore et al., 2021; Siple et al., 2020; Thorson et al., 2018), we adopt an approach that explicitly considers how asynchrony and stability relate to one another within complex, dynamic, and hierarchical systems like fisheries portfolios and ecological communities (Link, 2018). In the context of a community comprising multiple species whose biomasses fluctuate through time, asynchrony (ϕ) can be expressed as the ratio of community biomass stability ($S_{\text{Community}}$) to species biomass stability (${\bar{S}}_{\text{Species}}$), where $S_{\text{Community}}$ is the inverse temporal variability (i.e., the inverse coefficient of variation = 1/CV) of community biomass, and ${\bar{S}}_{\text{Species}}$ the inverse of the biomass‐weighted average temporal variability among species: $\phi =S_{\text{Community}}/{\bar{S}}_{\text{Species}}$ (Thibaut & Connolly, 2013; Wang et al., 2019; Zhao et al., 2022). In this formulation, asynchrony ϕ must be ≥1, meaning that the stability of the community will always exceed species stability unless all species dynamics are perfectly synchronous (ϕ=1). This measure of asynchrony integrates two related processes whose effects can be partitioned mathematically: statistical averaging and compensation (Zhao et al., 2022).

Statistical averaging is the enhancement of community stability that occurs when individual species fluctuate independently of one another through time, thereby reducing variability of the community in aggregate (Doak et al., 1998; Link, 2018), and depends on the quantity (i.e., species richness) and evenness of the temporal variances among species (Zhao et al., 2022). For example, the contribution of statistical averaging to ϕ will be low in a community characterized by few species whose population dynamics are weakly correlated or uncorrelated yet exhibit large differences in the magnitude of temporal variance among taxa. This unevenness in variances is a common feature of communities, which tend to be highly skewed in abundances among species (Avolio et al., 2019) and where population variances tend to increase nonlinearly with their means (Anderson et al., 2013; Mikkelson et al., 2011). In contrast, compensation effects (CPE) also contribute to ϕ, but increase when species within a community negatively covary. That is, different species may dominate through time, ensuring the steady temporal provision of ecosystem properties by a rotating group of constituents. CPE can occur in ecosystems due to species interactions (Del Río et al., 2017), temporal segregation in life history (Georgian & Wallace, 1983), and/or the differential responses of species to their environments (Brown et al., 2016).

Although asynchrony among populations of multiple species within a community—or analogously among stock harvest dynamics within a fishing portfolio—is expected to stabilize aggregate system properties via the above mechanisms, the relationship between asynchrony among fish populations and harvest stability depends on both ecological and social factors (Moore et al., 2021; Oken et al., 2021). For instance, asynchrony in interannual stock population dynamics can enhance the stability of dependent fishery harvest portfolios by allowing commercial fishers to maintain effort by switching among individual stocks as productivities change (Hilborn et al., 2003; Moore et al., 2021; Nesbitt & Moore, 2016; Oken et al., 2021; Schindler et al., 2010). Similarly, within‐year compensatory dynamics among stocks may allow fishers to exploit stocks over a longer period as harvestable biomass remains accessible to fishers for longer (Nesbitt & Moore, 2016; Oken et al., 2021; Schindler et al., 2010).

The stability imparted to the fishery harvest portfolio by asynchrony is also mediated by numerous regulatory and economic factors that affect how fishers allocate effort. For example, the harvest of a particular stock may be limited by management regulations (e.g., inability to attain licenses to target a particular stock, updates to regional catch quotas, or seasonal/area closures; Oken et al., 2021) or the potentially prohibitive costs associated with acquiring additional gear, licenses, or quota that may limit fisher diversification (Holland & Kasperski, 2016; Kasperski & Holland, 2013). Additionally, fishers may choose to specialize their portfolios by targeting particular stocks when market conditions are favorable (Anderson et al., 2017; Ward et al., 2018; White & Scheld, 2024). In these and similar scenarios, harvest portfolio stability may arise from regulatory or economic forces mediating how fishing effort is distributed across stocks rather than from the stability derived from natural asynchrony.

**FIGURE 1:**
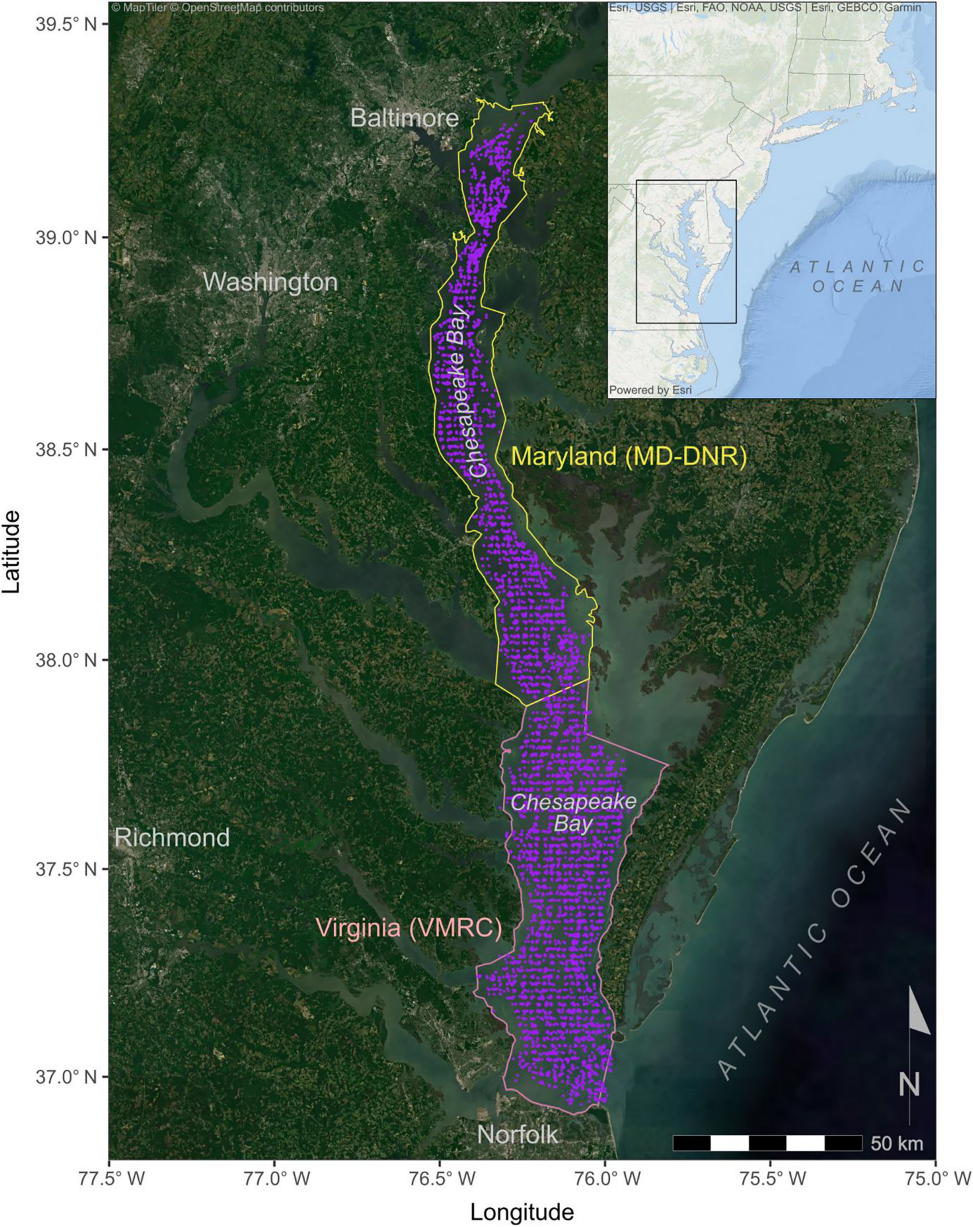
Map of Chesapeake Bay and its location along the U.S. East Coast (inset). Purple points are the locations of ChesMMAP survey trawls (2002–2018), and the yellow polygon shows the region of Maryland within which the commercial harvests used in Maryland analyses occurred. Commercial fishing within this region is managed by the Maryland Department of Natural Resources (MD‐DNR). Similarly, the pink polygon shows the region of Virginia where commercial harvests used in Virginia analyses occurred. Commercial fishing within this region is managed by the Virginia Marine Resources Commission (VMRC).

We explored these concepts within communities of demersal finfish and associated commercial fisheries in the mainstem of Chesapeake Bay, USA (Figure 1). The Chesapeake Bay is among the largest and most economically and ecologically valuable estuaries in the world and supports numerous high‐value commercial fisheries (Sanchirico et al., 2008). Many demersal fishes targeted by commercial fisheries in Chesapeake Bay are seasonally migratory, moving into or out of the bay mainstem into tributaries or onto the adjacent continental shelf in colder months (Buchheister et al., 2013; Schonfeld et al., 2022), and the seasonal timing of commercial fishing has adapted to these movement patterns. Fisheries management measures for migratory species are determined by state, interstate, and/or federal fishery management organizations through species‐specific management plans, although regulatory implementation and enforcement in state waters like the Chesapeake Bay is left to individual states. In the area of Chesapeake Bay considered in this study (hereafter, “the Bay”) there are two distinct management regions: in the northern mainstem (Figure 1, yellow), management falls to the Maryland (MD) state Department of Natural Resources (MD‐DNR), and in the southern mainstem (Figure 1, pink) to the commonwealth of Virginia (VA) via the Virginia Marine Resources Commission (VMRC).

The composition of demersal finfish communities, and ultimately commercial harvest portfolios, differs between the fresh‐mesohaline northern mainstem and the southern mainstem that opens to the Atlantic Ocean (Harding et al., 2019; Lefcheck et al., 2014). Demersal finfish community biomass in the northern mainstem is typically dominated by white perch (*Morone americana*) and striped bass (*Morone saxatilis*), and in the southern mainstem, by striped bass, Atlantic croaker (*Micropogonias undulatus*), spot (*Leiostomus xanthurus*), and summer flounder (*Paralichthys dentatus*) (Buchheister et al., 2013). The relatively low estuarine diversity, seasonally asynchronous dynamics among demersal finfishes, and well‐documented regulatory structuring along a prominent geographic and environmental gradient make Chesapeake Bay an ideal test case for evaluating how asynchrony among populations of exploited species relates to asynchrony and stability in dependent fisheries harvest portfolios.

**FIGURE 2:**
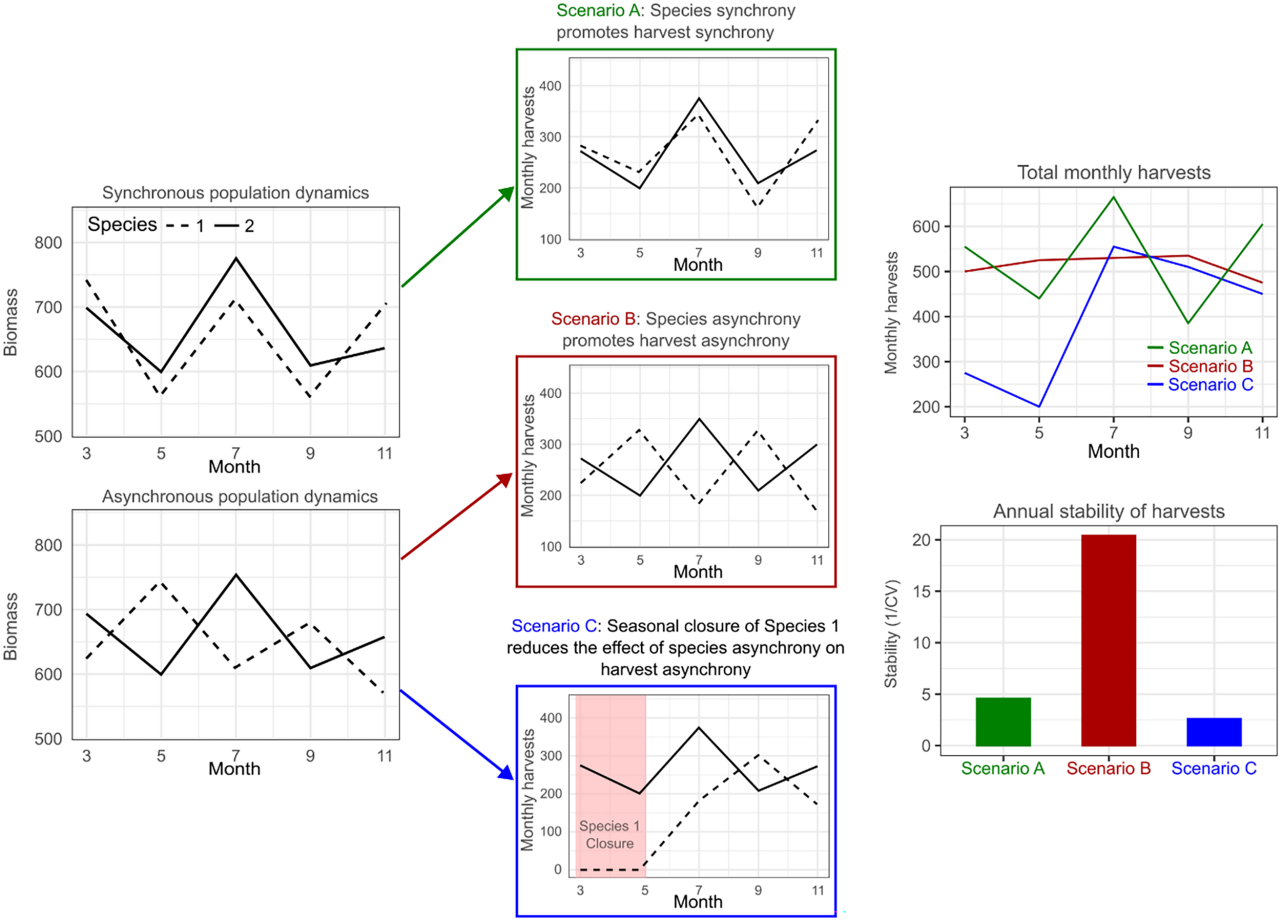
Conceptual diagram to visualize hypothetical relationships between asynchrony in the population dynamics of two harvested species (left column) and asynchrony in commercial harvests (center columns). Total monthly harvests from each hypothetical scenario are shown in the top right, and the resulting temporal stability of each harvest portfolio under the different scenarios is shown to the bottom right. Synchronous population dynamics of the two species lead to synchronous harvest, leading to low stability (Scenario A, green). Asynchronous population dynamics of the two species may lead to asynchronous harvest and high stability (Scenario B, red), but seasonal closure of one species reduces the effect of species asynchrony on harvest asynchrony and reduces stability (Scenario C, blue).

We hypothesized that asynchronous seasonal migrations among species would promote harvest asynchrony and therefore stabilize fisheries yields and that this stabilizing effect would be mediated by seasonal harvest closures. We expected that the influence of such regulatory measures would be particularly evident in MD, where the valuable commercial striped bass fishery (Table 1) is closed during the spring spawning season (March–May) (while no similar closure exists for striped bass in VA). To test our hypotheses, we partitioned asynchrony into compensatory and statistical averaging effects (SAE) among species' populations and among harvests of species, and related these quantities using structural equation models (SEMs). Additionally, given that target diversification has been shown to promote the stability (Anderson et al., 2017; Ward et al., 2018) and magnitude (White & Scheld, 2024) of annual income for individual fishers and fishing communities (Cline et al., 2017), we extended our analysis to evaluate the relationships between within‐year harvest portfolio stability, harvest portfolio economic stability, and overall portfolio value across harvests in MD and VA. We expected that the within‐year stability of harvest value and overall value of the harvest portfolio would be greater in years where yields were more temporally stable following seasonal asynchrony in species dynamics (Figure 2, scenario B).

**TABLE 1:** Species of demersal finfish considered in our analysis by region (Maryland [MD] or Virginia [VA]).

Region	Common name	Scientific name	Avg. annual harvests (kg, 2002–2018)	Avg. annual dockside value (USD, 2002–2018)
MD	Striped bass	*Morone saxatilis*	554,372	$3,031,511
MD	White perch	*Morone americana*	162,971	$266,940
MD	Atlantic croaker	*Micropogonias undulatus*	77,677	$106,049
MD	Channel catfish	*Ictalurus punctatus*	87,480	$106,442
MD	Gizzard shad	*Dorosoma cepedianum*	54,250	$17,153
VA	Atlantic croaker	*Micropogonias undulatus*	1,489,750	$2,215,735
VA	Spot	*Leiostomus xanthurus*	554,203	$1,231,253
VA	Striped bass	*Morone saxatilis*	304,782	$1,561,843

*Note*: Average annual harvests (kg) and average annual dockside value (January 2010 dollars; adjusted using the Bureau of Labor Statistics CPI‐U) reflect commercial fishing harvests from vessels operating in the Bay mainstem between 2002 and 2018 (see Figure 1 for the spatial footprint of commercial harvest data).

## METHODS

### Overview of the study design

To test the hypothesis that within‐year asynchrony among populations of commercially harvested demersal finfish species (hereafter *species asynchrony*) in the Chesapeake Bay was positively related to within‐year asynchrony among species harvests (*harvest asynchrony*), we developed a 17‐year (2002–2018) monthly (five months per year) time series of species‐specific biomass estimates (biomass indices) for six species commonly harvested by commercial fisheries in the MD and VA regions of the Bay mainstem (Table 1). We used these monthly time series to calculate within‐year species asynchrony and its components—*species statistical averaging* and *species compensation effects*—separately for each region.

We then used the total monthly harvests of these species from the same regions of the Bay in MD and VA (Figure 1) to calculate within‐year harvest asynchrony and its components, *harvest statistical averaging* and *harvest compensation effects*. Next, we related species statistical averaging and CPE to harvest statistical averaging and CPE using SEMs. In VA, we integrated an annual index of fishing effort across species into the SEM as a mediating covariate, testing the hypothesis that effort was driven by species statistical averaging (representing the evenness of biomass dynamics among harvested species) and contributed to *species harvest stability* (representing the inverse of an average of within‐year harvest variabilities among species). In MD, we developed an index of seasonal harvest diversification to test the hypothesis that harvest diversification during the striped bass fishing closure promoted harvest asynchrony.

**FIGURE 3:**
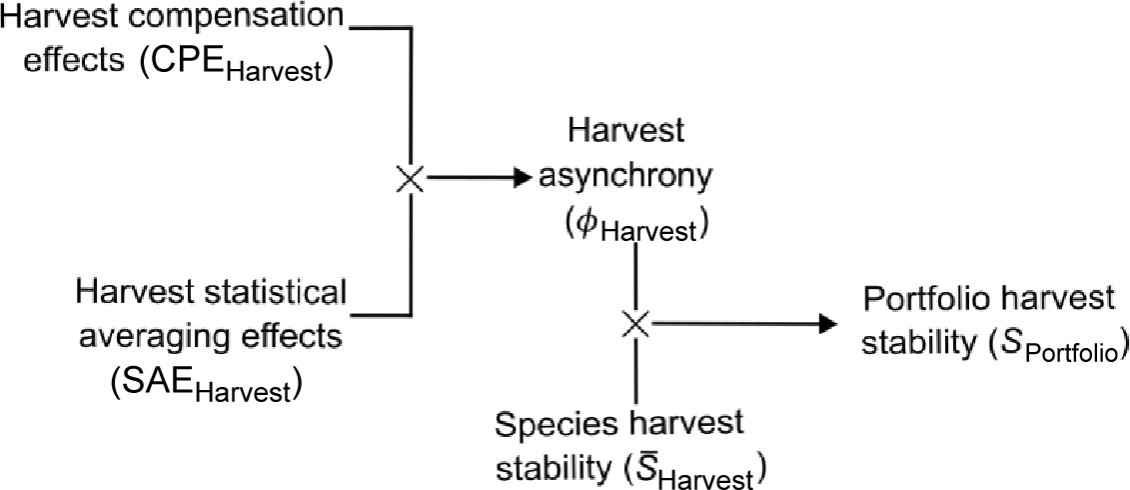
Mathematical relationships within the stability‐asynchrony partitioning framework, where quantities linked by × indicate a multiplicative relationship equating to the variable indicated by arrows.

Lastly, to evaluate how harvest asynchrony promoted total harvest yield and harvest value, and the within‐year stability of these metrics, we summed harvests (in kg) and harvest values (in USD) across species to create monthly indices of total harvest biomass and value. We used these indices to calculate the within‐year stability of total catch biomass (*portfolio harvest stability*), the within‐year stability of total catch value (*portfolio value stability*), and the annual sums of these monthly indices: *portfolio yield* and *portfolio value*, respectively. See Table 2 for definitions of asynchrony and stability‐related terms and Figure 3 for their mathematical relationships.

**TABLE 2:** Definitions of asynchrony and stability terms, their abbreviations, and mathematical formulas.

Term	Abbreviation	Formula	Description
Portfolio harvest stability (all months)	$S_{\text{Portfolio},L}$	${\left(\frac{\sigma_{R}}{\mu_{R}}\right)}^{-1}$	The stability of total harvests across all species and months.
Portfolio harvest stability (five months)	$S_{\text{Portfolio},S}$	${\left(\frac{\sigma_{R}}{\mu_{R}}\right)}^{-1}$	The stability of total harvests across all species during the five months when biomass estimates were available.
Species harvest stability	${\bar{S}}_{\text{Harvest}}$	${\left(\sum_{i}^{n}{\mathrm{CV}}_{i}\times \frac{\mu_{i}}{\mu_{R}}\right)}^{-1}$	The inverse of weighted‐average species harvest variability. We calculated this index for the five months when biomass estimates were available.
Asynchrony	ϕ	$\frac{\sum_{i}\sigma_{i}}{\sigma_{R}}$	A measure of the stabilizing effects of compensation and statistical averaging, either among harvests of multiple stocks $\left(\phi_{\text{Harvest}}\right)$ or among the biomass dynamics of multiple species $\left(\phi_{\text{Species}}\right)$.
Statistical averaging effect	SAE	$\frac{\sum_{i}\sigma_{i}}{\sqrt{\sum_{i}\sigma_{i}^{2}}}$	The enhancement of harvest or community stability following independent fluctuations among species harvests $\left(\mathrm{SA}E_{\text{Harvest}}\right)$ or population dynamics $\left(\mathrm{SA}E_{\text{Species}}\right)$.
Compensation effect	CPE	$\frac{\sqrt{\sum_{i}\sigma_{i}^{2}}}{\sigma_{R}}.$	The enhancement of harvest or community stability following negatively correlated dynamics among species harvests $\left(\mathrm{CP}E_{\text{Harvest}}\right)$ or population dynamics $\left(\mathrm{CP}E_{\text{Species}}\right)$.

### Bottom trawl survey and commercial harvest data

To characterize demersal finfish population dynamics, we derived within‐year biomass indices from the Chesapeake Bay Multispecies Monitoring and Assessment Program (ChesMMAP) bottom trawl survey (Latour et al., 2023). During 2002–2018, ChesMMAP cruises surveyed the entire Bay mainstem five times annually (targeting March, May, July, September, and November), sampling 300–400 locations per year. However, surveys in some month–year combinations did not occur, and in others sample coverage was relatively sparse (detailed below). The survey followed a stratified random design within three depth ranges (3.0–9.1 m, 9.1–15.2 m, and >15.2 m; Buchheister et al., 2013).

We estimated biomass indices for demersal finfishes that were both well sampled by the bottom trawl survey (see Buchheister et al., 2013) and well represented in regional harvest portfolios. To arrive at this latter characterization, we summed harvest yield by region and species across years, ranked these totals from highest to lowest, took the cumulative sum of ranked yields, and then selected the species that contributed to the top 90th percentile of cumulative yields (Table 1).

We focused our analysis on demersal finfish species and so did not consider two species that are particularly important to the Bay fishing economy, that is, blue crab and Atlantic menhaden. Although blue crabs are targeted by commercial fishers and adequately sampled by ChesMMAP trawl gear in the northern MD mainstem (Bilkovic et al., 2016), data from commercially licensed finfish and crab fishers in MD suggest that fishers tend to specialize in the harvest of either finfishes or crab. For example, the percentage of licensees reporting both crab and finfish commercial harvests in the MD Bay between 2012 and 2018 ranged from 12% to 15% (J. Baxter, personal communication, 2025), and fishers in VA with blue crab licenses also tend to be specialized (White & Scheld, 2024). Additionally, ChesMMAP trawls did not frequently occur within the shallow nearshore habitats where blue crab harvests primarily occur in VA waters (Figure 1, Bilkovic et al., 2016). Likewise, we did not consider Atlantic menhaden in our analysis due to the poor catchability of this schooling, pelagic species by ChesMMAP bottom trawl gear, and the fact that the stock is largely fished by a single company specializing in this species (Cuker, 2020).

**FIGURE 4:**
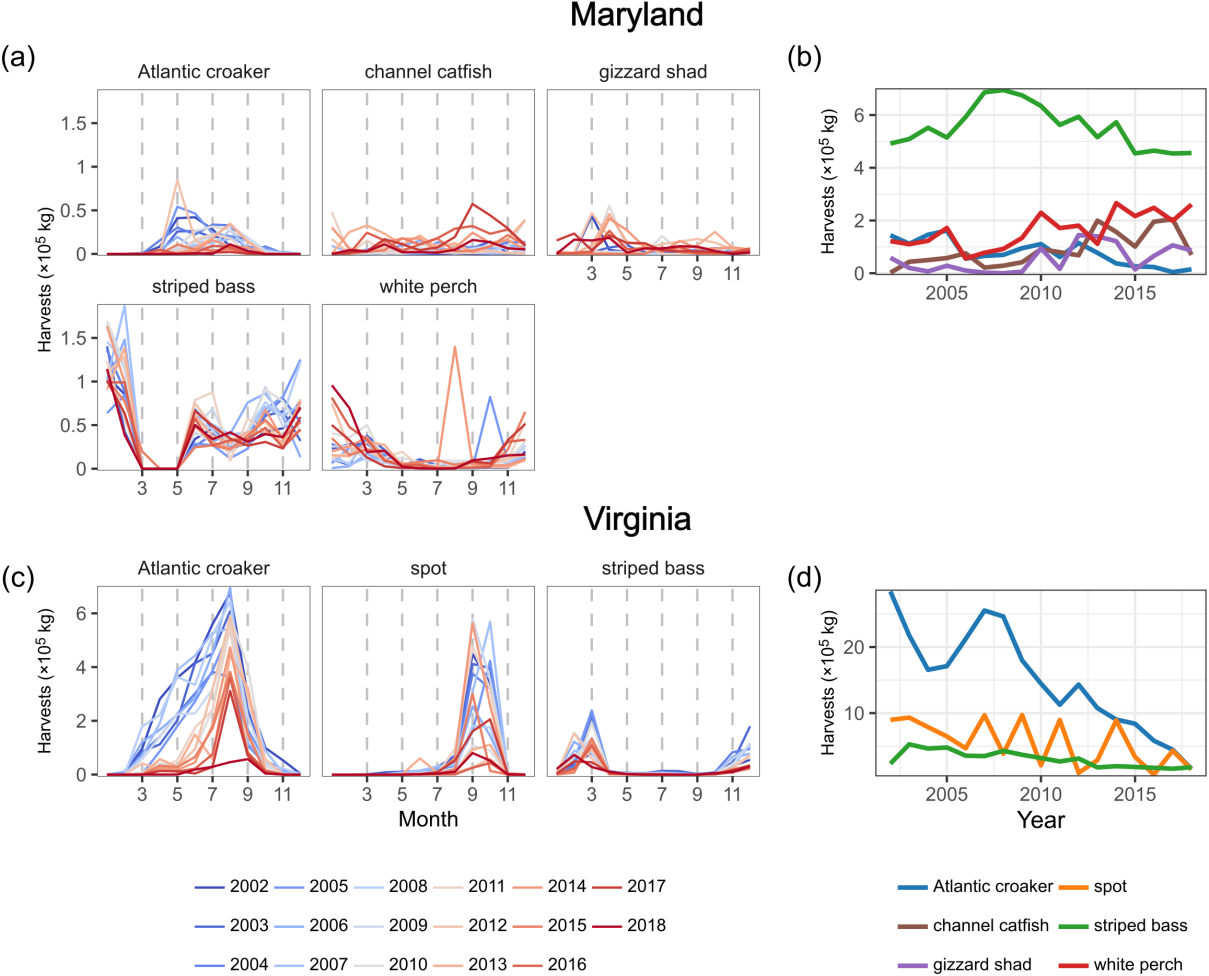
Seasonal patterns and interannual trends in mainstem commercial fisheries landings. Left: Within‐year time series of commercial fishing harvests in Maryland (a) and Virginia (c), where each line corresponds to a different year. Note that in Maryland the striped bass fishery is closed during March–May, and in Virginia the Atlantic croaker fishery is closed January 1–15, the spot fishery is closed December 8–April 15, and the striped bass fishery is closed January 1–16. Vertical dashed lines in (a) and (c) correspond to the months where biomass indices for each species were estimated (see *Methods*). Right: Total annual harvests of each species are shown for Maryland (b) and Virginia (d).

We evaluated the dynamics of demersal finfish harvests in the regions of MD and VA that overlapped spatially with ChesMMAP surveys (Figure 1) using monthly harvest biomass and value data provided by MD‐DNR and VMRC (Figure 4). The specific VMRC reporting areas from which harvest data were drawn included the Chesapeake Bay “upper west” (CBUW), “upper east” (CBUE), “lower west” (CBLW), “lower east” (CBLE), and “general” (CBG). The MD‐DNR reporting areas included the “southern” (code 029), “South Central” (027), and “North Central” (025) areas of the MD mainstem. Monthly harvest data from these areas were aggregated to reflect state‐specific spatial scales (Figure 1). We also received data on fishing effort in the form of the number of trips per month that fishers encountered each species in both regions. We created a combined annual index of trips across species within each region. This index was calculated by averaging the total number of trips for each species that occurred during the months when ChesMMAP surveys occurred (described below). Trip data in MD were available starting in 2005, whereas trip data in VA began in 2002.

To understand how the closure of the striped bass fishery impacted the stability and value of the portfolio in MD, we created an index called the seasonal harvest ratio (SHR). The SHR was the ratio of the average monthly harvests occurring during the striped bass fishery closure to the average monthly harvests occurring outside the striped bass fishery closure. When the SHR was high, harvests of species other than striped bass compensated for the absence of striped bass harvests during the closure. Lastly, to explore the mechanisms between harvest timing and the components of harvest asynchrony, we calculated the average month of harvest for each species and year, weighted by monthly harvests. For both the SHR and harvest timing indices, only fishing data from months overlapping with the ChesMMAP survey were used, recognizing that this survey comprises the primary fishing season in the Bay.

### Biomass index modeling

We used generalized additive mixed models (GAMMs) with spatial and spatiotemporal Gaussian Markov random fields to develop within‐year biomass indices for finfish species in MD and VA waters. We pursued this approach rather than simple aggregated CPUE to account for spatiotemporal variation in the density of species throughout the study period. We modeled biomass data with the Tweedie observation error distribution and a log link (Tweedie, 1984). All models included a categorical predictor with levels corresponding to the five target sampling months for the ChesMMAP survey. To account for interannual variability in month‐year predictions, we included factor‐smooth interactions (bs = “fs” in the R package mgcv [Wood, 2017]) using numeric month by year. For the spot biomass model, we included a thin‐plate regression smoother for the numeric year–month combination (Wood, 2017), and for the gizzard shad and channel catfish models, we included categorical predictors for year factors. These decisions improved convergence and residual behavior for these models over alternatives.

There were four instances where cruises did not occur in 2002–2018 (out of 85 possible; 4.7%). In fitting each model, we also excluded trawls collected from regions where a given species was not found throughout all surveys (Commander et al., 2022). This reduced the number of observations available for model fitting, especially for species whose distributions were limited to the northern mainstem (white perch, gizzard shad, and channel catfish). However, the inclusion of smooth terms allowed us to interpolate reasonable biomass estimates representing typical seasonal densities for each species during these periods and for when there were relatively few observations (e.g., in 3 of 85 month–year combinations, there were fewer than 30 trawls available for fitting the white perch model). To account for survey sampling locations and extend the indices over the area of species occurrence, we calculated area‐weighted biomass indices by predicting from each model over a grid with 6.25 km^2^ cells derived from a convex hull of trawls from the ChesMMAP survey (limited to regions where species occurred) and summed these predictions within months. The model for species besides spot, gizzard shad, and channel catfish can be represented as

(1) $$\begin{array}{l} E\left[y_{s,t}\right]\;=\mu_{s,t},\\ \mu_{s,t}\;=\exp\left(\alpha_{\text{season}}+f_{\text{year}}\left(\text{month}\right)+\omega_{s}+\epsilon_{s,t}\right),\\ \omega_{s}\;˜\mathrm{MVN}\left(0,\Sigma_{\omega }\right),\\ \epsilon_{s,t}\;˜\mathrm{MVN}\left(0,\Sigma_{\epsilon }\right), \end{array}$$

where $\mu_{s,t}$ is the mean at location *s* and time *t*, $\alpha_{\text{season}}$ is an overall intercept for each target month, $f_{\text{year}}\left(\text{month}\right)$ is the numeric month–year factor‐smooth interaction with common wiggliness, $\omega_{s}$ is a spatial random field value (varies by space but is constant with time), and $\epsilon_{s,t}$ is a spatiotemporal random field value that is independent for each year. The Gaussian random fields were approximated as Gaussian Markov random fields using the Stochastic Partial Differential Equation (SPDE) approach (Lindgren et al., 2011) with covariance matrices (i.e., inverse precision matrices) $\Sigma_{\omega }$ and $\Sigma_{\epsilon }$, each constrained by a Matérn covariance function (Cressie & Huang, 1999). We fitted these models in R (R Core Team, 2024) using the package sdmTMB (Anderson et al., 2024) via maximum marginal likelihood (see Appendix S1: Section S1 and supplemental R scripts in Hardison, 2025 for further details and visualizations of biomass indices with CIs).

### Partitioning stability and asynchrony and hypothesis testing

We applied the stability and asynchrony partitioning framework of Thibault and Connolly (2013; who considered the related quantities of variability and synchrony) that is commonly used in ecological contexts (Lamy et al., 2019; Wang et al., 2019) both to the communities of species harvested by commercial fisheries in the Bay and to the harvest portfolios in MD and VA (Figure 3, Table 2). When applied to the fishery harvest portfolio, the portfolio harvest stability is the inverse of total harvest variability, given by

(2) $$S_{\text{Portfolio}}={\left(\frac{\sigma_{R}}{\mu_{R}}\right)}^{-1},$$

where for region *R* (MD or VA), $\sigma_{R}$ is the SD of monthly total harvests within the year and $\mu_{R}$ is the temporal mean of monthly total harvests within the year. Portfolio harvest stability $\;\left(S_{\text{Portfolio}}\right)$ is the product of harvest asynchrony ($\phi_{\text{Harvest}}$) and species harvest stability (${\bar{S}}_{\text{Harvest}}$), with the latter term given by

(3) $${\bar{S}}_{\text{Harvest}}={\left(\sum_{i}^{n}{\mathrm{CV}}_{i}\times \frac{\mu_{i}}{\mu_{R}}\right)}^{-1},$$

which is the inverse of the harvest‐weighted average of coefficients of variation (CV) across *n* species. Each stock in our analyses refers to a single species, so we call this term species harvest stability (the inverse of species harvest variability) to mirror the use of the term species variability in the ecological literature (e.g., Wang et al., 2019).

We calculated $S_{\text{Portfolio}}$ twice for each year and region: once corresponding to the five months for which biomass estimates were available, that is, $S_{\text{Portfolio},S}$, and again for all 12 months of the year, $S_{\text{Portfolio},L}$, where the subscripts S and L refer to “short” and “long,” respectively. We then directly related $S_{\text{Portfolio},S}$ and $S_{\text{Portfolio},L}$ within SEMs, testing the hypothesis that the effects of species asynchrony, calculated using five months of data, indirectly related to the stability of the harvest portfolio when calculated using all available harvest data. We calculated portfolio value stability as the inverse temporal variability of summed monthly harvest values using all 12 months of harvest data. Code for stability partitioning was adapted from Lamy et al. (2019).

We next partitioned species asynchrony $\left(\phi_{\text{Species}}\right)$ and harvest asynchrony $\left(\phi_{\text{Harvest}}\right)$ into SAE and CPE (Zhao et al., 2022). Continuing in the context of the harvest portfolio, harvest asynchrony is given by $\phi_{\text{Harvest}}=\frac{\sum_{i}\sigma_{i}}{\sigma_{R}}=\mathrm{SA}E_{\text{Harvest}}\times \mathrm{CP}E_{\text{Harvest}}.$ Here,

(4) $$\mathrm{SA}E_{\text{Harvest}}=\frac{\sum_{i}\sigma_{i}}{\sqrt{\sum_{i}\sigma_{i}^{2}}},$$

and $\mathrm{SA}E_{\text{Harvest}}$ is the ratio comparing the SD of portfolio harvests if species harvests were perfectly synchronous to the SD of portfolio harvests if species harvests were completely uncorrelated (Zhao et al., 2022). Harvest compensation $\left(\mathrm{CP}E_{\text{Harvest}}\right)$ is then

(5) $$\mathrm{CP}E_{\text{Harvest}}=\frac{\sqrt{\sum_{i}\sigma_{i}^{2}}}{\sigma_{R}},$$

where CPE is equivalent to the inverse of the square‐rooted variance ratio (Schluter, 1984; Zhao et al., 2022), a commonly used measure of synchrony/asynchrony in the ecological literature. $\mathrm{CP}E_{\text{Harvest}}>1$ indicates greater portfolio harvest stability than would be expected if species harvests fluctuated independently of one another (Zhao et al., 2022). We partitioned $\phi_{\text{Species}}$ identically to $\phi_{\text{Harvest}}$ (Table 2), and both $\phi_{\text{Harvest}}$ and $\phi_{\text{Species}}$ were calculated using the same five months of harvest or biomass data yearly (the five months during which ChesMMAP surveys occurred).

We used piecewise SEMs to relate asynchrony and stability indices across ecological and fishery systems (Lefcheck, 2016). SEMs allow for the modeling of complex relationships where variables can occur as both responses and predictors and thus are ideal for identifying indirect and cascading effects. The overall model is achieved by combining a series of linear regressions (sub‐models) into a single network that can be assessed for significance and goodness‐of‐fit, which we evaluated using the Fisher's *C* statistic (Shipley, 2013; Appendix S1: Table S1). Within each SEM, we evaluated sub‐models for residual independence, normality, and homogeneity of variances. When interannual residual autocorrelation was present, we modeled residual error as an AR(1) process using generalized least squares (Pinheiro et al., 2017). We did not include species or harvest asynchrony (ϕ) indices explicitly in SEMs, as ϕ is a derived quantity, that is, ϕ=CPE×SAE (Figure 3). Further, we fit two SEMs within each region to improve interpretability and to avoid relating derived quantities in the models and inflating statistical (not causal) dependence: one linking biological and fishery variables, and a second linking portfolio harvest stability and economic variables. SEMs were fitted in R using the piecewiseSEM package (Lefcheck, 2016). To contextualize these results, we further modeled interannual trends in ecological and fishery variables using linear and smoother terms using GAMs in mgcv (Wood, 2017). We accounted for autocorrelated errors in these trend models using generalized least squares with AR(1) or AR(2) error structures following visual inspection of residual autocorrelation patterns.

## RESULTS

### Contributors to asynchrony and asynchrony relationships between the community and fishing portfolio

**FIGURE 5:**
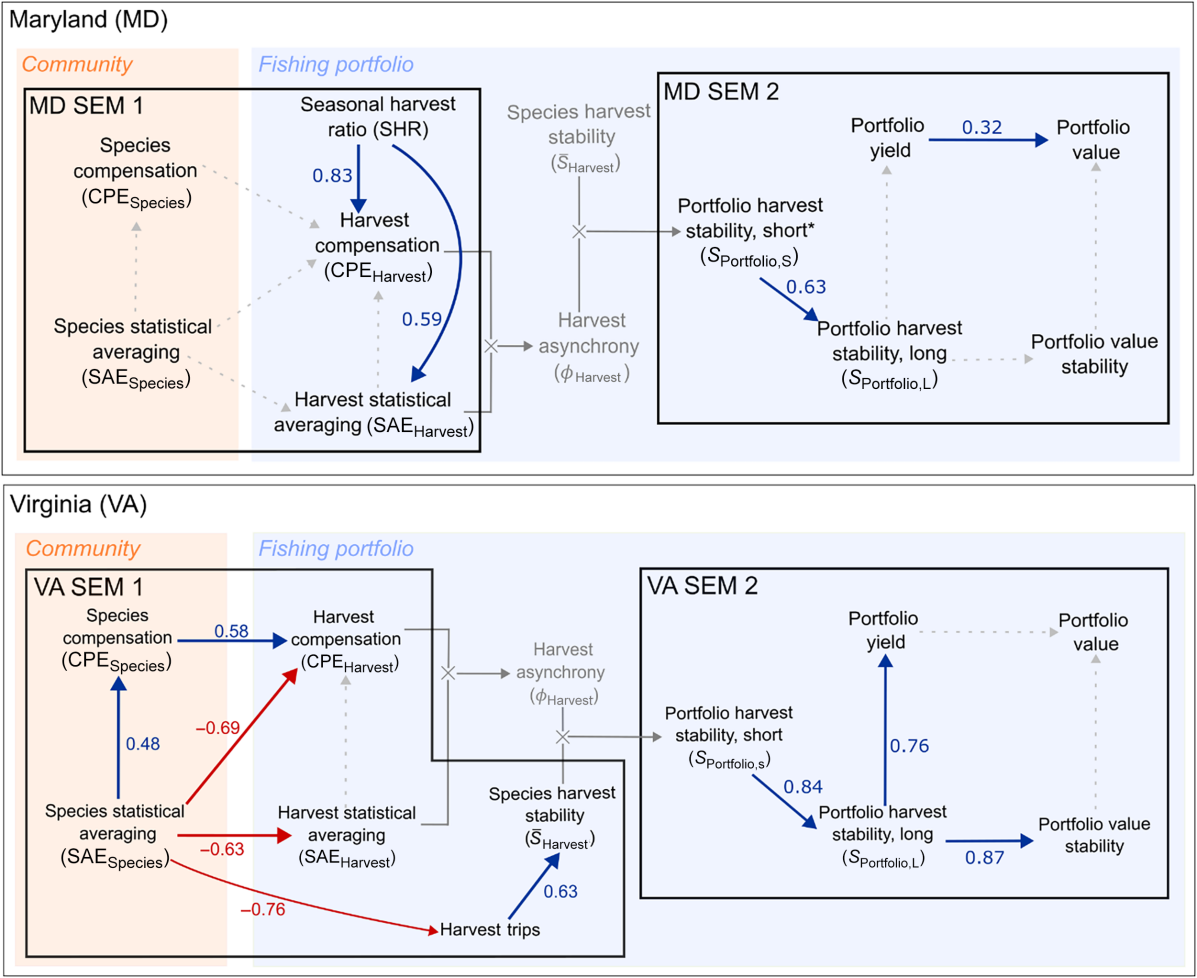
Linked path diagrams showing the relationships between asynchrony in the community (orange shading) and fishing portfolio (blue shading) in Maryland (top) and Virginia (bottom). Statistical relationships within black boxes were modeled using structural equation models (SEMs): Dark blue arrows indicate positive relationships (*p* < 0.1), red arrows negative relationships, and dotted gray lines no relationship. Numbers next to paths show standardized coefficients. The “*” in MD SEM 2 indicates the application of a log‐transformation to reduce residual nonlinearity in this sub‐model.

**FIGURE 6:**
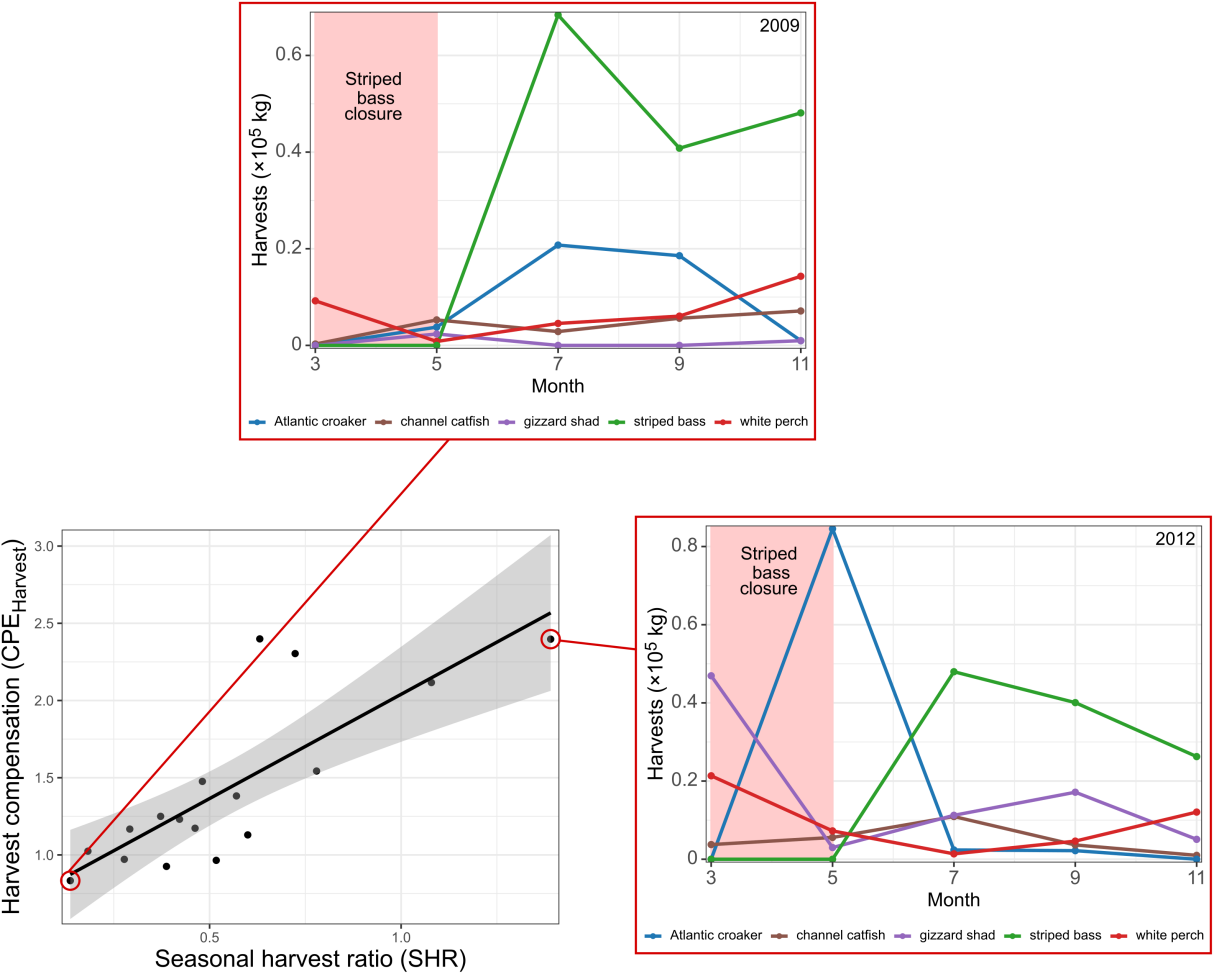
The relationship between the harvest compensation effect (CPE_Harvest_) and the seasonal harvest ratio (SHR) in Maryland (bottom left; *R* ^2^ = 0.64). Larger values of CPE_Harvest_ indicate greater stability of harvests via a portfolio effect, and CPE_Harvest_ > 1 indicates greater compensation in the harvest of species than would be expected if species harvests fluctuated independently of one another (see *Methods*). Larger values of SHR indicate greater compensation of harvest by other species during the Maryland striped bass fishery closure (see *Methods*). Red‐outlined panels show the Maryland harvest time series during years when the SHR was highest (right; 2012) and lowest (top; 2009). Note the relatively large harvests of Atlantic croaker, gizzard shad, and white perch in 2012 (high SHR), and low harvests of all species in 2009 (low SHR). Red shading denotes the seasonal striped‐bass fishery closure.

In MD, harvest compensation was positively associated with the SHR, the degree to which species other than striped bass contributed to harvests during the closed season (*p* = 0.001; Figure 5, MD SEM 1). In other words, diversification of harvests during the closure offset the loss of biomass in striped bass during this period of the year (Figure 6). The SHR was also positively associated with harvest statistical averaging (*p* = 0.02), indicating that harvests tended to be more temporally even across stocks when diversification occurred during the striped bass closure. However, we did not detect significant relationships between natural population fluctuations and harvest asynchrony in MD. Thus, asynchrony in MD fisheries harvests was facilitated more by management action than by the dynamics of exploited species' populations. Although we fitted a version of the MD SEM that integrated the fishing effort index as an additional covariate, this integration required that we remove three observations from all sub‐models to fit the model (valid trip data began in 2005 in MD), and there was no significant relationship between the trip index and other variables. For these reasons, we present the simpler analysis (MD SEM 1) that does not integrate the index of fishing effort.

**FIGURE 7:**
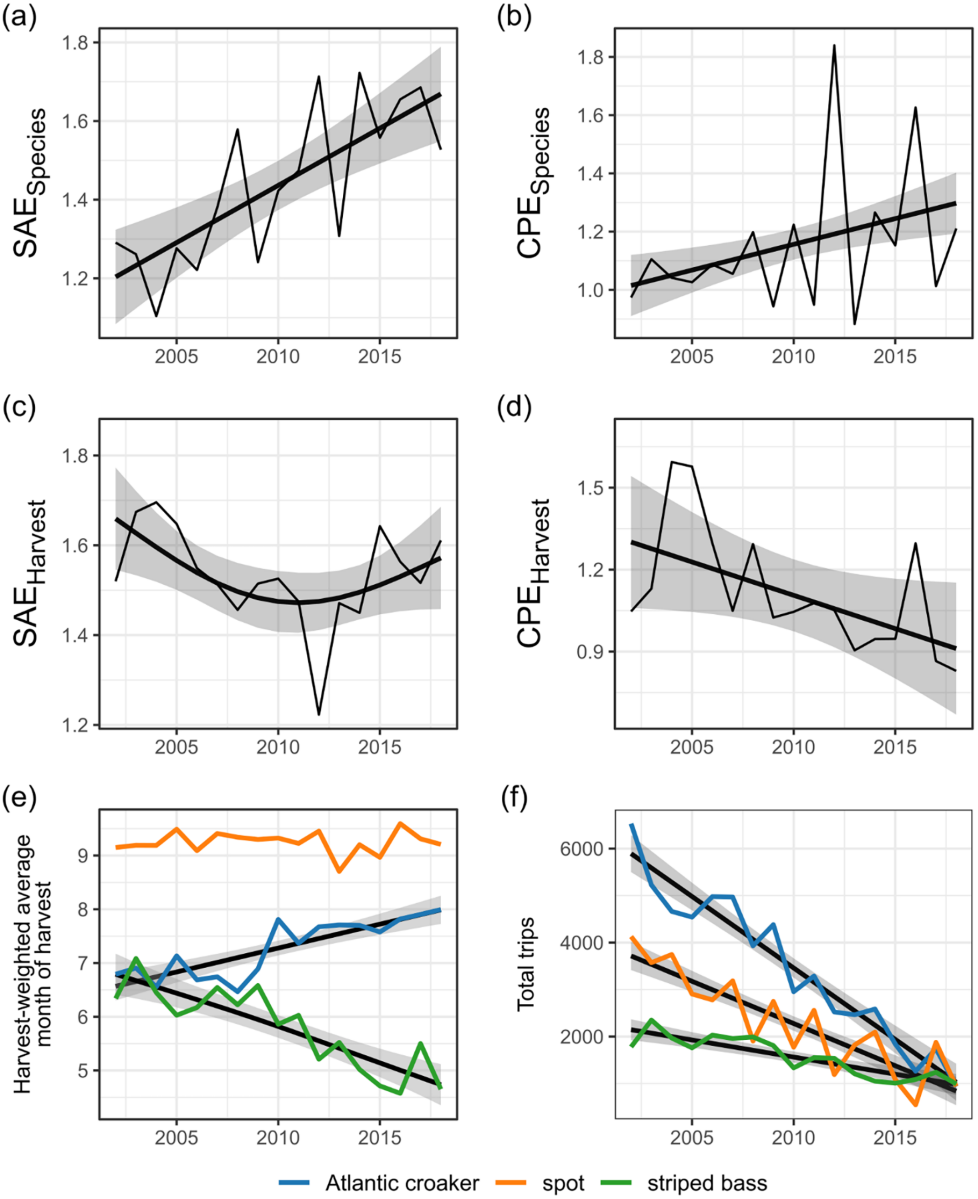
Interannual time series and trends for ecological variables in Virginia including species statistical averaging (SAE_Species_; a) and species compensation effects (CPE_Species_; b); fishery variables including harvest statistical averaging (SAE_Harvest_; c), harvest compensation effects (CPE_Harvest_; d), harvest‐weighted average month of harvest (e), and total annual trips by commercial fishermen (f). Thick black lines and gray shading show trends with 95% CIs, included when *p <* 0.1.

**FIGURE 8:**
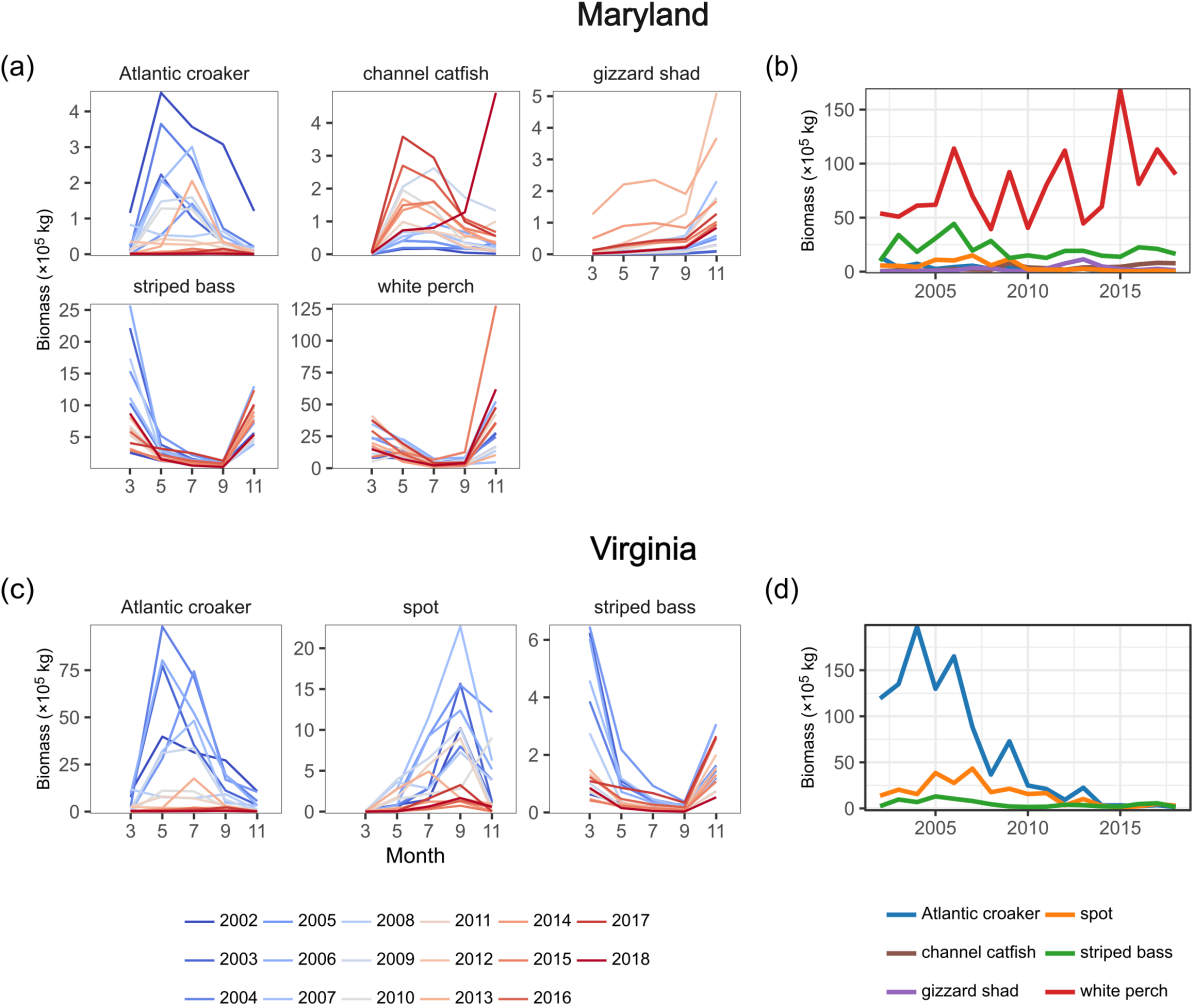
Seasonal patterns and interannual trends in estimated fish biomass. Left: Within‐year time series of fish biomasses in Maryland (a) and Virginia (c), where each line corresponds to a different year. Note that ChesMMAP surveys did not occur between November and March. Right: Total biomass of each species (summed across the five sampling months) are shown for Maryland (b) and Virginia (d).

In VA, increased species asynchrony over time arose from two sources: increased evenness over time following interannual declines in multiple dominant species (Figures 7a and 8d) and greater seasonal compensation that accompanied these biomass declines (Figure 7b). From 2002 to 2018, the biomass indices of the three species in VA (Atlantic croaker, spot, and striped bass) declined substantially (Figure 8c,d). However, these biomass declines were not equivalent in magnitude across species: average total annual biomass of Atlantic croaker declined by 92% between the 2002–2010 and 2011–2018 periods, whereas average spot biomass declined by 77%, and striped bass by 51%. After the substantial loss of Atlantic croaker biomass, the remaining (albeit substantially reduced) species biomasses became more evenly distributed, leading to an increased SAE (Figure 7a). The species SAE was then positively associated with species compensation (*p* = 0.002, Figure 5, VA SEM 1). In other words, following the steep declines in Atlantic croaker biomass from the first decade of the survey to the second, species population dynamics became more temporally even and seasonally compensatory, and therefore asynchronous.

**FIGURE 9:**
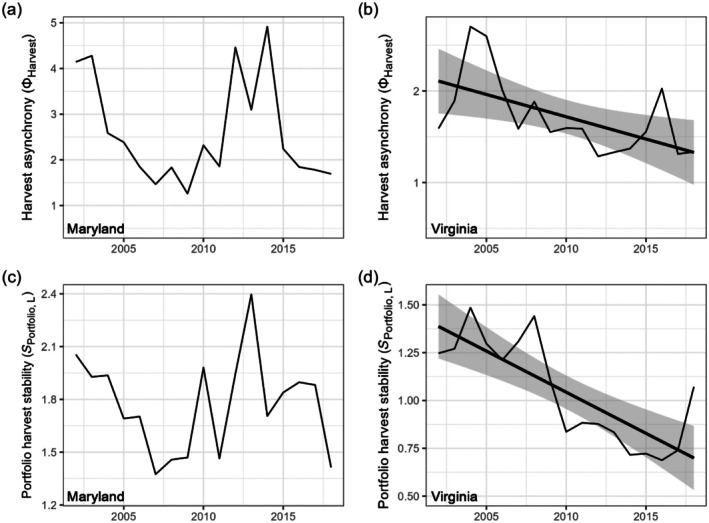
Time series of harvest asynchrony ($\phi_{\text{Harvest}}$) (a, b) and portfolio harvest stability (*S*_Portfolio,L_) (c, d) in Maryland (left column) and Virginia (right column). Here, $\phi_{\text{Harvest}}=\mathrm{CP}E_{\text{Harvest}}\times \mathrm{SA}E_{\text{Harvest}}$ so $\phi_{\text{Harvest}}$ reflects harvest asynchrony at the 5‐month time interval. Thick black lines and gray shading show trends with 95% CIs, included when *p <* 0.1.

Although species statistical averaging and compensation increased in the VA fish community over the study period, harvest compensation showed a weak negative trend (*p* = 0.079, Figure 7d, Appendix S1: Table S3), and was positively associated with species compensation ($\beta_{\mathrm{std}}=0.584,$ *p* = 0.053, Figure 5, VA SEM 1). This suggested that seasonal compensatory dynamics in population biomasses partly drove seasonal harvest compensation. However, the magnitude of the standardized partial effect of species statistical averaging on harvest compensation was greater than that of species compensation on harvest compensation and, importantly, opposite in sign ($\beta_{{\mathrm{SAE}}_{\mathrm{std}}}=-0.685$ vs. $\beta_{{\mathrm{CPE}}_{\mathrm{std}}}=0.584$). Thus, the relatively weak and stabilizing effect of seasonal species compensation on harvest compensation was negated by the destabilizing effects of declines in target species biomasses that led to increased species evenness (i.e., high species statistical averaging). Harvests from this less abundant and more even community became more temporally synchronous as a result (Figure 9b).

The negative effect of species statistical averaging (temporal evenness) on harvest compensation may have integrated the effects of declining overall biomass (Figure 8c,d) and subsequent reductions in effort (Figure 7f) on harvest timing (Figure 7e) that in turn contributed to harvest compensation. We found that Atlantic croaker harvests shifted later in the year, whereas striped bass harvests shifted earlier in the year (both *p* < 0.001, Figure 7e). However, only Atlantic croaker demonstrated a (negative) relationship between harvest timing and harvest compensation (*p* = 0.014, Appendix S1: Figure S1). When we repeated the analysis after calculating the harvest timing index using only the months when ChesMMAP surveys occurred (to match the temporal resolution of the harvest compensation index), we found that, in addition to the negative relationship between Atlantic croaker harvests and harvest compensation, the timing of spot harvests was negatively associated with harvest compensation (*p* = 0.019). Overall, these results suggest that harvest timing, particularly of Atlantic croaker which shifted over the study period, was a key contributor to harvest compensation and asynchrony.

### Harvest stability and economic performance

In MD (Figure 5, MD SEM 2), we found that portfolio harvest stability quantified at the same temporal resolution as the population biomass indices (i.e., *S*_Portfolio,S_) was positively associated with portfolio harvest stability quantified using all months of harvest data (*S*_Portfolio,L_). This finding shows that harvest asynchrony, induced by diversification (Figure 6) and quantified at the temporal resolution of the ChesMMAP survey, was positively associated with harvest stability when all months of data were integrated into the harvest stability measure (*S*_Portfolio,L_). However, portfolio harvest stability across all months was not linked to portfolio value stability, and in turn, portfolio value stability was not linked to portfolio value (Figure 5, MD SEM 2). In other words, harvest diversification by fishers buffered the stability of regional portfolio yield against losses due to regulatory measures, but this stabilizing effect did not lead to economic stability. Instead, we found that portfolio value stability was positively associated with the stability of within‐year striped bass harvest value (*p =* 0.005, Appendix S1: Figure S2), the dominant contributor to portfolio value (Table 1). A similar positive association arose for white perch harvest value stability, but this species contributed far less to portfolio value overall (Appendix S1: Figure S2, Table 1). Lastly, we identified a strong positive relationship between portfolio yield and value (*p* < 0.001), showing that years with larger harvests across months were associated with greater portfolio value across months (Figure 5, MD SEM 2).

In VA (Figure 5, VA SEM 2), we also identified a positive link between portfolio harvest stability at the 5‐ and 12‐month temporal resolutions. However, portfolio harvest stability (*S*_Portfolio,L_) declined substantially over the study period (Figure 9d, Appendix S1: Table S3, *p* < 0.001) following diminished harvest compensation (Figure 7d) and ultimately harvest asynchrony (Figure 9b). An additional contributor to the decline in portfolio harvest stability was a decline in fishing effort across stocks (Figures 5 and 7f, VA SEM 1) that led to reduced species harvest stability. In other words, the loss of species biomass—primarily of Atlantic croaker—was associated with lower fishing effort and more temporally synchronous harvests throughout the year, increasing within‐year temporal variability and decreasing the contributions of statistical averaging. Although portfolio harvest stability was positively associated with portfolio value stability and portfolio yield (Figure 5, VA SEM 2), this benefit was increasingly unrealized as harvest stability declined over time. Further, we did not find a relationship between portfolio value stability and portfolio value.

## DISCUSSION

Asynchronous dynamics among populations or species harvested by fisheries can promote stability in overall fish harvest over time (e.g., Schindler et al., 2010), but understanding the links between population asynchrony and harvest asynchrony is challenged by the many interacting ecological, social, and economic factors that mediate how fishing effort is distributed among stocks. We approached this problem by analyzing 17 years of data from fisheries‐independent community surveys and fisheries landings across one of the largest, most productive, and most economically valuable estuaries in the world, the Chesapeake Bay. We found that within‐year asynchrony in Chesapeake Bay finfish harvests was enhanced by harvest diversification following seasonal fishing closures (in MD) and seasonal compensatory dynamics among species (in VA). However, while species compensation was a positive contributor to harvest compensation in VA, this effect was counteracted by the negative effect of species statistical averaging on harvest compensation, reflecting changes in fishing patterns following declines in the biomass‐dominant species, primarily Atlantic croaker. Additionally, we found that fishing effort declined in response to this loss of biomass, ultimately contributing to reduced species and portfolio harvest stability despite a more even community in recent years. In neither region did we identify relationships between portfolio value and portfolio value stability, but in VA we found that portfolio harvest stability was positively associated with portfolio value stability. These findings demonstrated that promoting or conserving asynchronously fluctuating exploited natural resources can enhance the within‐year economic stability of dependent systems like fisheries, but that these stabilizing effects can be compromised by the total availability and variability of harvested species in the context of environmental fluctuations, management regulations, and human behavior.

Our application of this framework to a coupled social‐ecological system adds clarity to the linkages between species asynchrony—a process that is well known for stabilizing community dynamics in theoretical (Thibaut & Connolly, 2013; Wang & Loreau, 2016), experimental (Ma et al., 2021; Schnabel et al., 2021), and natural (Lamy et al., 2019; Muraina et al., 2021; Rogers & Schindler, 2008) systems—and the stability of dependent human systems. Several studies have explored similar relationships between community dynamics and harvest asynchrony, including in fisheries (Moore et al., 2021; Nesbitt & Moore, 2016; Oken et al., 2021; Schindler et al., 2010), and our findings build on this research by treating the populations harvested by fisheries and their associated harvests as belonging to hierarchically structured portfolios that are linked by asynchrony. By conceptualizing these systems separately, we identified the specific components of asynchrony that contributed to the stability of associated fishery harvest portfolios.

Cross‐system asynchrony relationships were limited in MD, where harvest asynchrony was related to the adaptive response of fishers to management strategies limiting access to the valuable striped bass fishery during part of the year. During the closure, fishers landed alternative species like Atlantic croaker and white perch, and harvested gizzard shad primarily as bycatch. Harvests of these species promoted harvest asynchrony and ultimately enhanced the stability of the harvest portfolio across the year. However, the stabilizing effect of diversification did not promote portfolio value stability due to the comparatively lower value of these other species being harvested during the seasonal closure compared to striped bass. Despite the closure, portfolio value stability was largely driven by the stability of striped bass harvest value, suggesting that this portfolio was diversified more in terms of catch than of economic return.

Interannual declines in the biomass of Atlantic croaker, and to a lesser extent spot, in VA (also see Buchheister et al., 2013; Schonfeld et al., 2022) promoted species statistical averaging that was associated with reduced fishing effort, harvest asynchrony, and species and portfolio harvest stability. When the community was dominated by these species in the first decade of the survey (leading to low evenness and low statistical averaging), effort was high and harvests tended to be more seasonally compensatory, promoting species and portfolio harvest stability. However, as Atlantic croaker and spot were lost and the species remaining in the system appeared more even, fishing effort and harvests declined and became more synchronous, contributing to declining species and portfolio harvest stability from year to year. Effort has been further reduced by a recent lack of entry by younger participants into VA's small‐scale commercial fisheries (White & Scheld, 2022). Thus, the capacity of the fishery system to realize greater stability in the latter years of the survey was compromised by the overall lack of fishable biomass and fewer fishers. Promisingly, ChesMMAP surveys since 2018 have reported higher abundances of spot and Atlantic croaker (Ralph et al., 2025), with this rebound being more pronounced for spot than Atlantic croaker.

The mechanisms through which species statistical averaging (or changes in evenness) impacted fishing effort and harvest compensation were likely driven by fish ecology and/or fisher behavior. Substantial declines in the abundance of Atlantic croaker during the spring and summer months during the latter period of the survey almost certainly contributed to smaller harvests during those months, resulting in a shift in harvest timing toward autumn. As the timing of Atlantic croaker harvests shifted, there was likely greater overlap between Atlantic croaker harvests and the directed spot fishery that occurs primarily in the autumn, reducing harvest compensation within a largely diminished harvest portfolio. Coincident with Atlantic croaker biomass declines were decreases in Atlantic croaker body size (Bonzek et al., 2019), reducing marketability (L. Gillingham, personal communication, 2024), fishing effort and, ultimately, in harvests.

Declines in Atlantic croaker effort would have also contributed to declines in spot effort during the summer months when Atlantic croaker and spot are frequently harvested together using the same gear. Although we did not explore shifting phenological dynamics in the current analysis, an alternative explanation for declines in harvest compensation could be that phenological shifts in the timing of different species utilizing the Bay led to altered harvest timing. Such shifts are not unheard of in the Bay: for instance, cobia (*Rachycentron canadum*) have been documented migrating into the Bay earlier in the season during years when water temperatures are warmer, impacting when cobia are targeted by recreational fishers (Crear et al., 2020).

Target diversification by individual fishers (Anderson et al., 2017; Kasperski & Holland, 2013; Ward et al., 2018; White & Scheld, 2024) and fishing communities (Cline et al., 2017; Sethi et al., 2014) tends to enhance fishing revenue stability, and how evenly harvest revenues are distributed across stocks is an important mediator of this portfolio effect (Cline et al., 2017). In MD, where the value of the regional harvest portfolio was dominated by the contributions of a single stock, we found that portfolio value stability depended less on the harvest dynamics of other stocks in the portfolio and more on the value stability of the dominant stock (striped bass). In VA, harvest value was on average more evenly distributed among species (Table 1) such that species and harvest compensation promoted portfolio harvest stability and portfolio value stability, although this effect was diminished in recent years with the loss of significant contributors to biomass. However, we did not uncover a relationship between portfolio value stability and portfolio value in either region. This result may be a consequence of how we quantified portfolio value stability, which can be identical between years that have different magnitudes of portfolio value so long as the ratio of the within‐year temporal mean to temporal SD of portfolio value remains the same.

One additional diversification opportunity that may prove important for providing stability to commercial fishers in the Bay is the emerging market for blue catfish (*Ictalurus furcatus*). Blue catfish is an invasive species whose abundances have increased dramatically in Bay tributaries in recent years; annual Bay harvests of blue catfish now frequently exceed those of striped bass (Fabrizio et al., 2021). Government support for a blue catfish fishery is also growing, evidenced by recent legislative efforts from the governments of MD and VA supporting the development of a commercial fishery (Maryland General Assembly, 2021; Virginia General Assembly, 2023), although barriers such as ex‐vessel price and consumer demand still exist (Scheld et al., 2024).

While blue catfish were rare in ChesMMAP surveys that occur in the mainstem of the Bay and so were excluded from our analysis, the growing importance of this fishery raises a limitation of the presented analysis. First, the Bay is home to several species of finfishes and invertebrates that we did not include in our analysis. For instance, the Bay supports enormous blue crab fisheries that were valued across both states at 45–108 million USD per year (2002–2018; NMFS, 2024), and harvests of the pelagic Atlantic menhaden across the Bay are considerable (e.g., between 2001 and 2005, landings from the Atlantic menhaden reduction fishery averaged 109,020 mt annually, although this number has declined in more recent years following a reduction in the harvest cap to 51,000 mt) (Anstead et al., 2021). We did not consider these species in our analysis due to issues related to catchability, fisher diversification patterns, and spatial overlap between ChesMMAP and where harvests occur in the Bay. However, expanding the study region while integrating additional data sources into the spatiotemporal index standardization approach (such as in Grüss & Thorson, 2019) may allow for the consideration of these species in future analyses. Similar analyses could also be conducted for the economically critical recreational fishing industry in the Chesapeake Bay where 13 million angler trips are recorded annually (Scheld et al., 2024). Additionally, we assumed that the indices derived from spatiotemporal models and used within the stability partitioning framework were known without error, a potential pitfall (Brooks & Deroba, 2015) that could be addressed by propagating biomass index uncertainty through stability partitioning analyses using simulated indices from a parametric bootstrap (e.g., Thorson et al., 2024). Lastly, we assumed a unidirectional relationship between species abundances and species harvests, although harvesting will certainly influence the population dynamics of harvested species, particularly as this pressure is exerted over time (Arlinghaus et al., 2017; Moore et al., 2021). Such approaches are hindered by the difficulty of statistically disentangling such bidirectional relationships, but future analytical developments may yet allow for rigorous tests.

Ecological communities and the fisheries that depend on them share common features that can be leveraged to better understand how their emergent properties behave and relate to one another. In this work, we have evaluated how the emergent properties of asynchrony and stability relate between communities of demersal finfishes and their associated regional harvest portfolios, showing that harvest asynchrony and stability depend not only on species asynchrony but also on the management regimes and market forces that mediate how fishing effort is distributed among naturally fluctuating stocks and decadal changes in biomass availability. Continuing to make these cross‐system linkages explicit will facilitate a deeper understanding of how humanity benefits from the conservation of biological complexity and promote more responsible and sustainable use of natural resources to support human well‐being.

## CONFLICT OF INTEREST STATEMENT

The authors declare no conflicts of interest.

## Supporting information


Appendix S1:

## Data Availability

Data and code (Hardison, 2025) are available in Zenodo at https://doi.org/10.5281/zenodo.15995514.

## References

[eap70097-bib-0001] Anderson, S. C. , A. B. Cooper , and N. K. Dulvy . 2013. “Ecological Prophets: Quantifying Metapopulation Portfolio Effects.” Methods in Ecology and Evolution 4(10): 971–981.

[eap70097-bib-0073] Anderson, S. C. , E. J. Ward , P. A. English , L. A. K. Barnett , and J. T. Thorson . 2024. sdmTMB: An R Package for Fast, Flexible, and User‐Friendly Generalized Linear Mixed Effects Models with Spatial and Spatiotemporal Random Fields. bioRxiv. 10.1101/2022.03.24.485545.

[eap70097-bib-0003] Anderson, S. C. , E. J. Ward , A. O. Shelton , M. D. Adkison , A. H. Beaudreau , R. E. Brenner , A. C. Haynie , J. C. Shriver , J. T. Watson , and B. C. Williams . 2017. “Benefits and Risks of Diversification for Individual Fishers.” Proceedings of the National Academy of Sciences of the United States of America 114(40): 10797–10802. 28923938 10.1073/pnas.1702506114 PMC5635870

[eap70097-bib-0071] Anstead, K. A. , K. Drew , D. Chagaris , M. Cieri , A. M. Schueller , J. E. McNamee , A. Buchheister , et al. 2021. “The Path to an Ecosystem Approach for Forage Fish Management: A Case Study of Atlantic Menhaden.” Frontiers in Marine Science 8: 607657.

[eap70097-bib-0004] Arlinghaus, R. , J. Alós , B. Beardmore , K. Daedlow , M. Dorow , M. Fujitani , D. Hühn , et al. 2017. “Understanding and Managing Freshwater Recreational Fisheries as Complex Adaptive Social‐Ecological Systems.” Reviews in Fisheries Science & Aquaculture 25(1): 1–41.

[eap70097-bib-0005] Avolio, M. L. , E. J. Forrestel , C. C. Chang , K. J. La Pierre , K. T. Burghardt , and M. D. Smith . 2019. “Demystifying Dominant Species.” New Phytologist 223(3): 1106–1126. 30868589 10.1111/nph.15789

[eap70097-bib-0006] Bilkovic, D. M. , H. W. Slacum , K. J. Havens , D. Zaveta , C. F. Jeffrey , A. M. Scheld , D. Stanhope , K. Angstadt , and J. D. Evans . 2016. Ecological and Economic Effects of Derelict Fishing Gear in the Chesapeake Bay 2015/2016 Final Assessment Report (2016). Gloucester Point, VA: Viginia Institute of Marine Science, William & Mary. 10.21220/V54K5C.

[eap70097-bib-0007] Bonzek, C. F. , J. Gartland , D. J. Gouthier , and R. J. Latour . 2019. Data Collection and Analysis in Support of Single and Multispecies Stock Assessments in Chesapeake Bay: The Chesapeake Bay Multispecies Monitoring and Assessment Program (Project Number F‐130‐R‐14). Gloucester Point, VA: Viginia Institute of Marine Science, William & Mary. 10.25773/3v19-3f27.

[eap70097-bib-0008] Brooks, E. N. , and J. J. Deroba . 2015. “When “Data” Are Not Data: The Pitfalls of Post Hoc Analyses that Use Stock Assessment Model Output.” Canadian Journal of Fisheries and Aquatic Sciences 72(4): 634–641.

[eap70097-bib-0009] Brown, B. L. , A. L. Downing , and M. A. Leibold . 2016. “Compensatory Dynamics Stabilize Aggregate Community Properties in Response to Multiple Types of Perturbations.” Ecology 97(8): 2021–2033. 27859207 10.1890/15-1951.1

[eap70097-bib-0010] Buchheister, A. , C. F. Bonzek , J. Gartland , and R. J. Latour . 2013. “Patterns and Drivers of the Demersal Fish Community of Chesapeake Bay.” Marine Ecology Progress Series 481: 161–180.

[eap70097-bib-0011] Cline, T. J. , D. E. Schindler , and R. Hilborn . 2017. “Fisheries Portfolio Diversification and Turnover Buffer Alaskan Fishing Communities from Abrupt Resource and Market Changes.” Nature Communications 8(1): 1–7. 10.1038/ncomms14042 PMC5241831 28091534

[eap70097-bib-0012] Commander, C. J. , L. A. Barnett , E. J. Ward , S. C. Anderson , and T. E. Essington . 2022. “The Shadow Model: How and Why Small Choices in Spatially Explicit Species Distribution Models Affect Predictions.” PeerJ 10: e12783. 35186453 10.7717/peerj.12783 PMC8852273

[eap70097-bib-0013] Crear, D. P. , B. E. Watkins , M. A. Friedrichs , P. St‐Laurent , and K. C. Weng . 2020. “Estimating Shifts in Phenology and Habitat Use of Cobia in Chesapeake Bay under Climate Change.” Frontiers in Marine Science 7: 579135.

[eap70097-bib-0074] Cressie, N. , and H.‐C. Huang . 1999. “Classes of Nonseparable, Spatio‐Temporal Stationary Covariance Functions.” Journal of the American Statistical Association 94(448): 1330–1339. 10.1080/01621459.1999.10473885.

[eap70097-bib-0015] Cuker, B. E. 2020. Menhaden, the Inedible Fish that Most Everyone Eats. Diet for a Sustainable Ecosystem: The Science for Recovering the Health of the Chesapeake Bay and Its People, 95–106. Cham: Springer International Publishing.

[eap70097-bib-0016] Del Río, M. , H. Pretzsch , R. Ruíz‐Peinado , E. Ampoorter , P. Annighöfer , I. Barbeito , K. Bielak , et al. 2017. “Species Interactions Increase the Temporal Stability of Community Productivity in *Pinus sylvestris*–*Fagus sylvatica* Mixtures across Europe.” Journal of Ecology 105(4): 1032–1043.

[eap70097-bib-0017] Doak, D. F. , D. Bigger , E. Harding , M. Marvier , R. O'Malley , and D. Thomson . 1998. “The Statistical Inevitability of Stability‐Diversity Relationships in Community Ecology.” The American Naturalist 151(3): 264–276. 10.1086/286117 18811357

[eap70097-bib-0018] Fabrizio, M. C. , V. Nepal , and T. D. Tuckey . 2021. “Invasive Blue Catfish in the Chesapeake Bay Region: A Case Study of Competing Management Objectives.” North American Journal of Fisheries Management 41: S156–S166.

[eap70097-bib-0072] Food and Agriculture Organization of the United Nations (FAO). The State of World Fisheries and Aquaculture . 2024. Blue Transformation in Action, Vol. 2024. FAO. 10.4060/cd0683en.

[eap70097-bib-0019] Georgian, T. , and J. B. Wallace . 1983. “Seasonal Production Dynamics in a Guild of Periphyton‐Grazing Insects in a Southern Appalachian Stream.” Ecology 64(5): 1236–1248.

[eap70097-bib-0020] Grüss, A. , and J. T. Thorson . 2019. “Developing Spatio‐Temporal Models Using Multiple Data Types for Evaluating Population Trends and Habitat Usage.” ICES Journal of Marine Science 76(6): 1748–1761.

[eap70097-bib-0021] Harding, L. W. , M. E. Mallonee , E. S. Perry , W. D. Miller , J. E. Adolf , C. L. Gallegos , and H. W. Paerl . 2019. “Long‐Term Trends, Current Status, and Transitions of Water Quality in Chesapeake Bay.” Scientific Reports 9(1): 1–19. 31040300 10.1038/s41598-019-43036-6 PMC6491606

[eap70097-bib-0022] Hardison, S. 2025. “Seanhardison1/asynchrony_across_systems: V1.0.0 – Accompanying Materials for ‘Seasonal Asynchrony and Harvest Diversification Contribute to Demersal Finfish Fisheries Stability in Chesapeake Bay’.” v1.0.0, Zenodo. 10.5281/zenodo.15995514.40936222

[eap70097-bib-0023] Hilborn, R. , J.‐J. Maguire , A. M. Parma , and A. A. Rosenberg . 2001. “The Precautionary Approach and Risk Management: Can they Increase the Probability of Successes in Fishery Management?” Canadian Journal of Fisheries and Aquatic Sciences 58(1): 99–107.

[eap70097-bib-0024] Hilborn, R. , T. P. Quinn , D. E. Schindler , and D. E. Rogers . 2003. “Biocomplexity and Fisheries Sustainability.” Proceedings of the National Academy of Sciences of the United States of America 100(11): 6564–6568. 12743372 10.1073/pnas.1037274100 PMC164486

[eap70097-bib-0025] Holland, D. S. , and S. Kasperski . 2016. “The Impact of Access Restrictions on Fishery Income Diversification of US West Coast Fishermen.” Coastal Management 44(5): 452–463.

[eap70097-bib-0026] Kasperski, S. , and D. S. Holland . 2013. “Income Diversification and Risk for Fishermen.” Proceedings of the National Academy of Sciences of the United States of America 110(6): 2076–2081. 23341621 10.1073/pnas.1212278110 PMC3568353

[eap70097-bib-0027] Lamy, T. , S. Wang , D. Renard , K. D. Lafferty , D. C. Reed , and R. J. Miller . 2019. “Species Insurance Trumps Spatial Insurance in Stabilizing Biomass of a Marine Macroalgal Metacommunity.” Ecology 100(7): e02719. 31081945 10.1002/ecy.2719

[eap70097-bib-0028] Latour, R. J. , J. Gartland , and C. F. Bonzek . 2023. “Design and Redesign of a Bottom Trawl Survey in Chesapeake Bay, USA.” Frontiers in Marine Science 10: 1217792.

[eap70097-bib-0029] Lefcheck, J. S. 2016. “piecewiseSEM: Piecewise Structural Equation Modelling in R for Ecology, Evolution, and Systematics.” Methods in Ecology and Evolution 7(5): 573–579.

[eap70097-bib-0030] Lefcheck, J. S. , A. Buchheister , K. M. Laumann , M. A. Stratton , K. L. Sobocinski , S. T. Chak , T. R. Clardy , P. L. Reynolds , R. J. Latour , and J. E. Duffy . 2014. “Dimensions of Biodiversity in Chesapeake Bay Demersal Fishes: Patterns and Drivers through Space and Time.” Ecosphere 5(2): 1–48.

[eap70097-bib-0031] Levin, S. , T. Xepapadeas , A.‐S. Crépin , J. Norberg , A. De Zeeuw , C. Folke , T. Hughes , et al. 2013. “Social‐Ecological Systems as Complex Adaptive Systems: Modeling and Policy Implications.” Environment and Development Economics 18(2): 111–132.

[eap70097-bib-0032] Lindgren, F. , H. Rue , and J. Lindström . 2011. “An Explicit Link between Gaussian Fields and Gaussian Markov Random Fields: The Stochastic Partial Differential Equation Approach.” Journal of the Royal Statistical Society Series B: Statistical Methodology 73(4): 423–498.

[eap70097-bib-0033] Link, J. S. 2018. “System‐Level Optimal Yield: Increased Value, Less Risk, Improved Stability, and Better Fisheries.” Canadian Journal of Fisheries and Aquatic Sciences 75(1): 1–16.

[eap70097-bib-0035] Ma, F. , F. Zhang , Q. Quan , B. Song , J. Wang , Q. Zhou , and S. Niu . 2021. “Common Species Stability and Species Asynchrony Rather than Richness Determine Ecosystem Stability under Nitrogen Enrichment.” Ecosystems 24: 686–698.

[eap70097-bib-0036] Maryland General Assembly, Senate . 2021. “Senate Joint Resolution No. 4. 2021 Legislative Session.” https://mgaleg.maryland.gov/2021RS/chapters_noln/jr_1_sj0004T.pdf.

[eap70097-bib-0037] Mikkelson, G. M. , B. J. McGill , S. Beaulieu , and P. L. Beukema . 2011. “Multiple Links between Species Diversity and Temporal Stability in Bird Communities across North America.” Evolutionary Ecology Research 13(4): 361–372.

[eap70097-bib-0038] Moore, J. W. , B. M. Connors , and E. E. Hodgson . 2021. “Conservation Risks and Portfolio Effects in Mixed‐Stock Fisheries.” Fish and Fisheries 22(5): 1024–1040.

[eap70097-bib-0039] Muraina, T. O. , C. Xu , Q. Yu , Y. Yang , M. Jing , X. Jia , M. S. Jaman , et al. 2021. “Species Asynchrony Stabilises Productivity under Extreme Drought across Northern China Grasslands.” Journal of Ecology 109(4): 1665–1675.

[eap70097-bib-0040] Nesbitt, H. K. , and J. W. Moore . 2016. “Species and Population Diversity in Pacific Salmon Fisheries Underpin Indigenous Food Security.” Journal of Applied Ecology 53(5): 1489–1499.

[eap70097-bib-0041] NMFS (National Marine Fisheries Service) . 2024. Landings [Online Database]. Silver Spring, MD: NMFS. https://www.st.nmfs.noaa.gov/commercial-fisheries/commercial-landings/annual-landings/index.

[eap70097-bib-0042] Oken, K. L. , D. S. Holland , and A. E. Punt . 2021. “The Effects of Population Synchrony, Life History, and Access Constraints on Benefits from Fishing Portfolios.” Ecological Applications 31(4): e2307. 33604951 10.1002/eap.2307

[eap70097-bib-0043] Pinheiro, J. , D. Bates , S. DebRoy , D. Sarkar , S. Heisterkamp , B. Van Willigen , and R. Maintainer . 2017. “Package ‘nlme.’ Linear and Nonlinear Mixed Effects Models, Version, 3(1), 274.”

[eap70097-bib-0044] R Core Team . 2024. R: A Language and Environment for Statistical Computing. Vienna: R Foundation for Statistical Computing. https://www.R-project.org/.

[eap70097-bib-0045] Ralph, G. M. , J. Gartland , D. J. Gauthier , J. Gregg , and J. R. Latour . 2025. Data Collection and Analysis in Support of Single and Multispecies Stock Assessments in Chesapeake Bay: The Chesapeake Bay Multispecies Monitoring and Assessment Program (Project Number F‐130‐R‐20). Gloucester Point, VA: Viginia Institute of Marine Science, William & Mary.

[eap70097-bib-0046] Rogers, L. A. , and D. E. Schindler . 2008. “Asynchrony in Population Dynamics of Sockeye Salmon in Southwest Alaska.” Oikos 117(10): 1578–1586.

[eap70097-bib-0047] Sanchirico, J. N. , M. D. Smith , and D. W. Lipton . 2008. “An Empirical Approach to Ecosystem‐Based Fishery Management.” Ecological Economics 64(3): 586–596.

[eap70097-bib-0048] Scheld, A. M. , D. M. Bilkovic , S. Stafford , K. Powers , S. Musick , and A. G. Guthrie . 2024. “Valuing Shoreline Habitats for Recreational Fishing.” Ocean & Coastal Management 253: 107150.

[eap70097-bib-0049] Schindler, D. E. , R. Hilborn , B. Chasco , C. P. Boatright , T. P. Quinn , L. A. Rogers , and M. S. Webster . 2010. “Population Diversity and the Portfolio Effect in an Exploited Species.” Nature 465(7298): 609–612. 20520713 10.1038/nature09060

[eap70097-bib-0050] Schluter, D. 1984. “A Variance Test for Detecting Species Associations, with Some Example Applications.” Ecology 65(3): 998–1005.

[eap70097-bib-0051] Schnabel, F. , X. Liu , M. Kunz , K. E. Barry , F. J. Bongers , H. Bruelheide , A. Fichtner , et al. 2021. “Species Richness Stabilizes Productivity Via Asynchrony and Drought‐Tolerance Diversity in a Large‐Scale Tree Biodiversity Experiment.” Science Advances 7(51): eabk1643. 34919425 10.1126/sciadv.abk1643 PMC8682986

[eap70097-bib-0052] Schonfeld, A. J. , J. Gartland , and R. J. Latour . 2022. “Spatial Differences in Estuarine Utilization by Seasonally Resident Species in Mid‐Atlantic Bight, USA.” Fisheries Oceanography 31(6): 615–628.

[eap70097-bib-0053] Sethi, S. A. , M. Reimer , and G. Knapp . 2014. “Alaskan Fishing Community Revenues and the Stabilizing Role of Fishing Portfolios.” Marine Policy 48: 134–141.

[eap70097-bib-0054] Shipley, B. 2013. “The AIC Model Selection Method Applied to Path Analytic Models Compared Using Ad‐Separation Test.” Ecology 94(3): 560–564. 23687881 10.1890/12-0976.1

[eap70097-bib-0055] Siple, M. C. , T. E. Essington , L. A. Barnett , and M. D. Scheuerell . 2020. “Limited Evidence for Sardine and Anchovy Asynchrony: Re‐Examining an Old Story.” Proceedings of the Royal Society B: Biological Sciences 287(1922): 20192781. 10.1098/rspb.2019.2781 PMC7126059 32156216

[eap70097-bib-0056] Thibaut, L. M. , and S. R. Connolly . 2013. “Understanding Diversity–Stability Relationships: Towards a Unified Model of Portfolio Effects.” Ecology Letters 16(2): 140–150. 10.1111/ele.12019 PMC3588152 23095077

[eap70097-bib-0058] Thorson, J. T. , A. G. Andrews, III , T. E. Essington , and S. I. Large . 2024. “Dynamic Structural Equation Models Synthesize Ecosystem Dynamics Constrained by Ecological Mechanisms.” Methods in Ecology and Evolution 15(4): 744–755.

[eap70097-bib-0057] Thorson, J. T. , M. D. Scheuerell , J. D. Olden , and D. E. Schindler . 2018. “Spatial Heterogeneity Contributes More to Portfolio Effects than Species Variability in Bottom‐Associated Marine Fishes.” Proceedings of the Royal Society B 285(1888): 20180915. 30282649 10.1098/rspb.2018.0915 PMC6191698

[eap70097-bib-0059] Tilman, D. 1996. “Biodiversity: Population Versus Ecosystem Stability.” Ecology 77(2): 350–363.

[eap70097-bib-0060] Tilman, D. , C. L. Lehman , and C. E. Bristow . 1998. “Diversity‐Stability Relationships: Statistical Inevitability or Ecological Consequence?” The American Naturalist 151(3): 277–282. 10.1086/286118 18811358

[eap70097-bib-0061] Tweedie, M. C. 1984. “An Index which Distinguishes between some Important Exponential Families.” Statistics: Applications and New Directions: Proceedings. Indian Statistical Institute Golden Jubilee International Conference 579: 579–604.

[eap70097-bib-0062] Virginia General Assembly . 2023. “Senate Bill 897: Blue Catfish Processing, Flash Freezing, and Infrastructure Grant Program; Created. Acts of Assembly, Chapter 134.” 2023 Regular Session. Virginia Legislative Information System. https://legacylis.virginia.gov/cgi-bin/legp604.exe?231+ful+CHAP0134.

[eap70097-bib-0063] Wang, S. , T. Lamy , L. M. Hallett , and M. Loreau . 2019. “Stability and Synchrony across Ecological Hierarchies in Heterogeneous Metacommunities: Linking Theory to Data.” Ecography 42(6): 1200–1211.

[eap70097-bib-0064] Wang, S. , and M. Loreau . 2016. “Biodiversity and Ecosystem Stability across Scales in Metacommunities.” Ecology Letters 19(5): 510–518. 26918536 10.1111/ele.12582 PMC4825858

[eap70097-bib-0070] Ward, E. J. , S. C. Anderson , A. O. Shelton , R. E. Brenner , M. D. Adkison , A. H. Beaudreau , J. T. Watson , J. C. Shriver , A. C. Haynie , and B. C. Williams . 2018. “Effects of Increased Specialization on Revenue of Alaskan Salmon Fishers Over Four Decades.” Journal of Applied Ecology 55(3): 1082–1091.

[eap70097-bib-0065] White, S. B. , and A. M. Scheld . 2022. “Characterizing Changes in Participation and Diversification in Small‐Scale Fisheries of Virginia, USA.” Coastal Management 50(1): 3–28.

[eap70097-bib-0066] White, S. B. , and A. M. Scheld . 2024. “Assessing Diversification Behavior of Small‐Scale Commercial Fishers.” ICES Journal of Marine Science 81(3): 480–490.

[eap70097-bib-0075] Wood, S. N. 2017. Generalized Additive Models: An Introduction with R, 2nd ed. New York: Chapman and Hall/CRC. 10.1201/9781315370279.

[eap70097-bib-0068] Zhao, L. , S. Wang , R. Shen , Y. Gong , C. Wang , P. Hong , and D. C. Reuman . 2022. “Biodiversity Stabilizes Plant Communities through Statistical‐Averaging Effects Rather than Compensatory Dynamics.” Nature Communications 13(1): 7804. 10.1038/s41467-022-35514-9 PMC9759569 36528635

