# Supplementary material for: Seasonal asynchrony and harvest diversification contribute to demersal finfish fisheries stability in Chesapeake Bay
Source: Ecol Appl. 2025 Sep 11;35(6):e70097. doi: 10.1002/eap.70097 (PMC12426367; doi:10.1002/eap.70097)
Supplement: Supplementary file 1 — Appendix S1: [file EAP-35-e70097-s001.pdf]

Appendix S1

Supplement to: **Seasonal asynchrony and harvest diversification contribute to demersal finfish fisheries stability in Chesapeake Bay**

Ecological Applications

Authors: Sean B. Hardison, Jonathan S. Lefcheck, Shelby B. White, Maowei Liang, Y. Stacy Zhang, Christopher J. Patrick, Andrew M. Scheld, Robert J. Latour, F. Joel Fodrie, Sean C. Anderson, and Max C. N. Castorani

Section S1.

Additional information regarding SPDE mesh creation and biomass index model diagnostics

sdmTMB integrates SPDE matrices from R-INLA (Lindgren & Rue, 2015) with marginal log likelihood calculations and random effect integration via the Laplace approximation with TMB (Kristensen et al., 2015). Our 'mesh' for the SPDE calculations was constructed in R-INLA with an inner mesh near the data and an outer mesh further away from the data to reduce boundary effects (Lindgren & Rue, 2015). Our inner and outer meshes had an 'offset' of 3 km and 8 km from the data, maximum triangle edge lengths of 3 km and 15 km, and a minimum triangle edge length of 3 km.

We assessed model convergence by checking that the maximum absolute gradient of the marginal log likelihood with respect to all fixed effects was < 0.001 and the Hessian matrix was positive definite. We used Markov Chain Monte Carlo (MCMC) randomized quantile residuals to evaluate residual patterning using QQ-plots. These residuals are samples of the random effects

drawn from the joint posterior distribution of the fitted model while fixed effects are held at their maximum likelihood estimates (Anderson et al. 2022). See the folder /R/biomass_indices/ in the code repository (Hardison 2025; https://doi.org/10.5281/zenodo.15995514) for further documentation of biomass index modeling.

Table S1. Goodness of fit measures for SEMs. See Fig. 4 in main text for reference.

Model	Fisher's *C*	*P*	df
MD SEM 1	1.33	0.86	4
MD SEM 2	5.49	0.86	10
VA SEM 1	20.08	0.22	16
VA SEM 2	8.31	0.60	10

Table S2. Statistics from linear and non-linear models (GAM) evaluating interannual temporal trends in within-year species and harvest statistical averaging ($SAE_{Species}$ and $SAE_{Harvests}$) and within-year species and harvest compensation effects ($CPE_{Species}$ and $CPE_{Harvests}$) in the Virginia portion of Chesapeake Bay over 2002-2018.

Linear model statistics

Term	Trend (SAE/CPE year^{-1})	Std. error	T statistic	P value	Error structure
$SAE_{Species}$	0.029	0.007	4.458	< 0.001	iid
$CPE_{Species}$	0.018	0.006	3.055	0.008	AR(2)
$CPE_{Harvests}$	-0.024	0.013	-1.887	0.079	AR(1)

Non-linear model statistics

Term	EDF	Ref. DF	F statistic	P value	Error structure
**$SAE_{Harvests}$**	**1.84**	1.97	2.84	0.077	iid

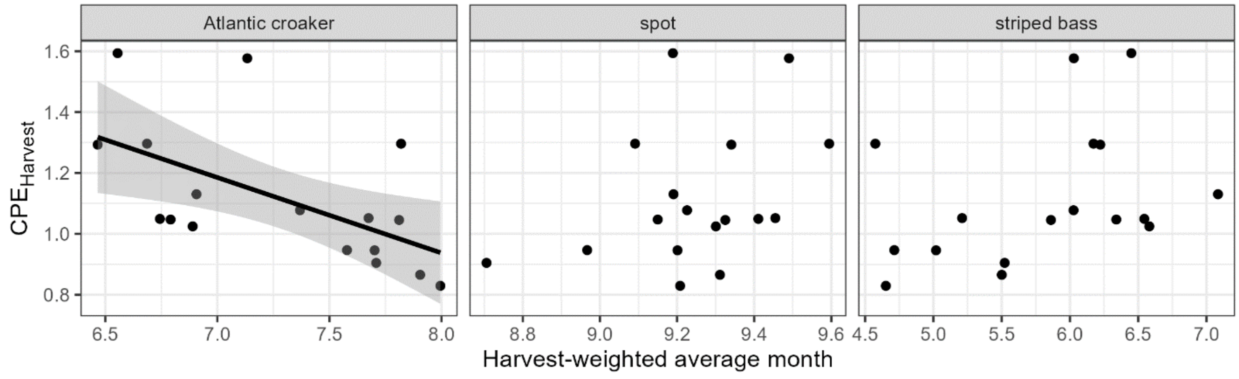

Figure S1. Relationships between harvest compensation and the weighted-average month of harvests (weights are harvests). We only found the relationship between the timing of Atlantic croaker harvests and harvest compensation significant ($P = 0.014$).

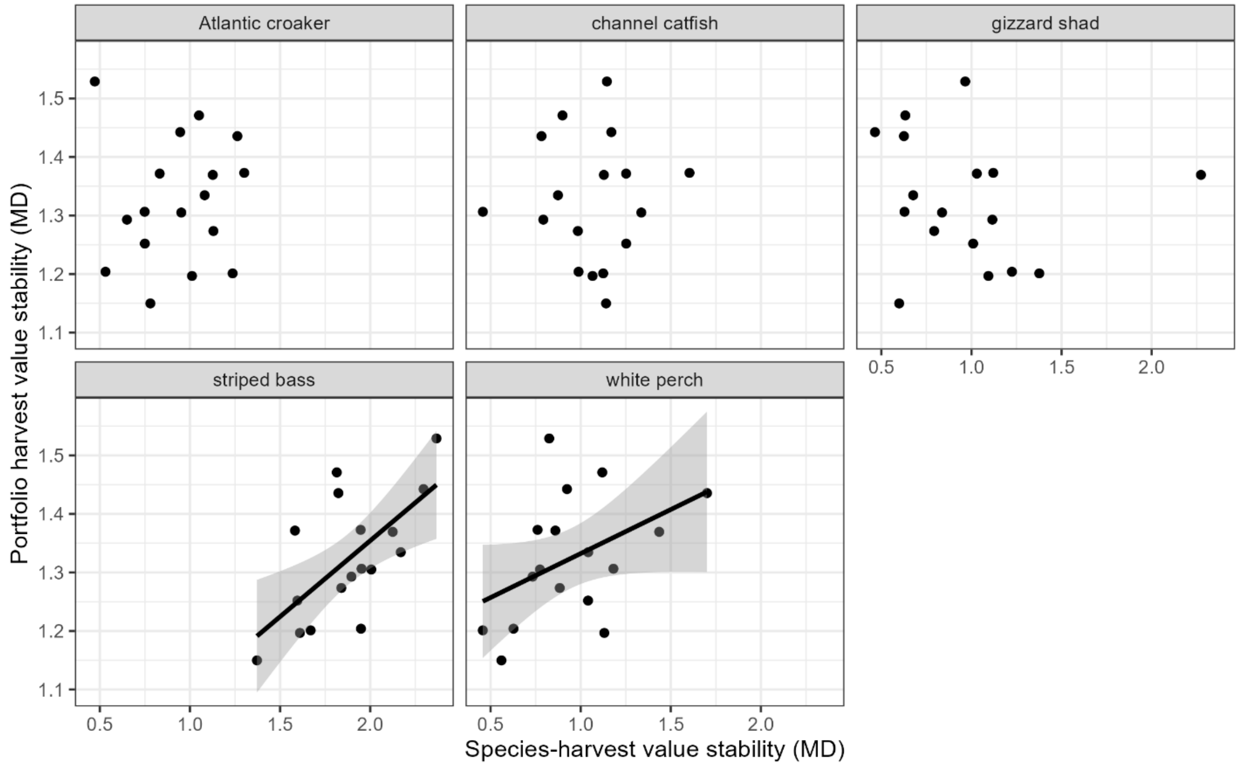

Figure S2. Portfolio harvest value stability in relation to species-specific harvest value stability in MD. Species-specific harvest value stability is 1/CV of within-year harvest values.

Table S3. Statistics from linear models evaluating interannual temporal trends in within-year harvest asynchrony ($\phi_{Harvest}$) and portfolio harvest stability ($S_{Portfolio,L}$) in Maryland and Virginia regions of the Chesapeake Bay over 2002-2018.

**Region**	**Term (stability year $^{-1}$)**	**Trend**	**Std. error**	**T statistic**	**P value**	**Error structure**
Maryland	$\phi_{Harvest}$	-0.05	0.058	-0.816	0.427	iid
Virginia	$\phi_{Harvest}$	-0.05	0.018	-2.760	0.015	iid
Maryland	$S_{Portfolio, L}$	-0.002	0.014	-0.158	0.877	iid
Virginia	$S_{Portfolio, L}$	-0.043	0.008	-5.130	0.000	iid

References

Anderson, S.C., E.J. Ward, P.A. English, L.A.K. Barnett, J.T. Thorson. 2024. sdmTMB: an R package for fast, flexible, and user-friendly generalized linear mixed effects models with spatial and spatiotemporal random fields. *bioRxiv* 2022.03.24.485545; doi: https://doi.org/10.1101/2022.03.24.485545

Hardison, S. (2025). seanhardison1/asynchrony_across_systems: v1.0.0 - Accompanying materials for "Seasonal asynchrony and harvest diversification contribute to demersal finfish fisheries stability in Chesapeake Bay" (v1.0.0). Zenodo. https://doi.org/10.5281/zenodo.15995514

Lindgren, F., & Rue, H. (2015). Bayesian spatial modelling with R-INLA. *Journal of Statistical Software*, *63*(19).

Kristensen, K., Nielsen, A., Berg, C. W., Skaug, H., & Bell, B. (2015). TMB: automatic differentiation and Laplace approximation. *arXiv Preprint arXiv:1509.00660.*
